# Immunity and Coagulation in COVID-19

**DOI:** 10.3390/ijms252011267

**Published:** 2024-10-19

**Authors:** Piotr P. Avdonin, Maria S. Blinova, Anastasia A. Serkova, Lidia A. Komleva, Pavel V. Avdonin

**Affiliations:** Koltzov Institute of Developmental Biology RAS, ul. Vavilova, 26, 119334 Moscow, Russia; ppavdonin@gmail.com (P.P.A.);

**Keywords:** COVID-19, inflammation, thrombotic microangiopathies, complement, coagulation, immunothrombosis

## Abstract

Discovered in late 2019, the SARS-CoV-2 coronavirus has caused the largest pandemic of the 21st century, claiming more than seven million lives. In most cases, the COVID-19 disease caused by the SARS-CoV-2 virus is relatively mild and affects only the upper respiratory tract; it most often manifests itself with fever, chills, cough, and sore throat, but also has less-common mild symptoms. In most cases, patients do not require hospitalization, and fully recover. However, in some cases, infection with the SARS-CoV-2 virus leads to the development of a severe form of COVID-19, which is characterized by the development of life-threatening complications affecting not only the lungs, but also other organs and systems. In particular, various forms of thrombotic complications are common among patients with a severe form of COVID-19. The mechanisms for the development of thrombotic complications in COVID-19 remain unclear. Accumulated data indicate that the pathogenesis of severe COVID-19 is based on disruptions in the functioning of various innate immune systems. The key role in the primary response to a viral infection is assigned to two systems. These are the pattern recognition receptors, primarily members of the toll-like receptor (TLR) family, and the complement system. Both systems are the first to engage in the fight against the virus and launch a whole range of mechanisms aimed at its rapid elimination. Normally, their joint activity leads to the destruction of the pathogen and recovery. However, disruptions in the functioning of these innate immune systems in COVID-19 can cause the development of an excessive inflammatory response that is dangerous for the body. In turn, excessive inflammation entails activation of and damage to the vascular endothelium, as well as the development of the hypercoagulable state observed in patients seriously ill with COVID-19. Activation of the endothelium and hypercoagulation lead to the development of thrombosis and, as a result, damage to organs and tissues. Immune-mediated thrombotic complications are termed “immunothrombosis”. In this review, we discuss in detail the features of immunothrombosis associated with SARS-CoV-2 infection and its potential underlying mechanisms.

## 1. Introduction

Coronavirus disease 2019 (COVID-19), caused by the enveloped single-stranded (+)RNA virus of the Coronaviridae family, Severe acute respiratory syndrome-related coronavirus 2 (SARS-CoV-2), has become the largest human pandemic of the current century. At the time of writing, according to the Worldometer resource (https://www.worldometers.info/coronavirus/ (accessed on 13 April 2024)), more than 700 million cases of the disease and more than 7 million deaths have been identified worldwide. The clinical manifestations of COVID-19 are diverse and can vary greatly [1]. As a rule, COVID-19 is mild, affects only the upper respiratory tract, and is accompanied by symptoms such as fatigue, fever, and dry cough [2]. However, in some cases, SARS-CoV-2 infection provokes the development of severe complications requiring hospitalization and intensive care [3]. The progression of COVID-19 can lead to viral pneumonia, acute respiratory distress syndrome (ARDS), damage to organs and organ systems, and the development of multiple-organ failure (MOF) with a high risk of death [2,3]. It has also been reported that in 10% of cases, there is a long-term (more than 12 weeks) debilitating course of COVID-19, the so-called long COVID or post-COVID-19 syndrome, which significantly reduces the quality of life and physical condition of patients [4]. Although many efforts are being made to establish the mechanisms of pathogenesis of COVID-19, they still remain unclear.

Clinical studies of patients conducted at the very beginning of the pandemic in China showed a violation of the blood coagulation function, leading to the development of venous thromboembolism, in patients with COVID-19 [5]. These observations were confirmed by further studies, which showed that COVID-19-associated coagulopathy (CAC) contributes to morbidity and mortality in patients infected with SARS-CoV-2 [5,6,7,8,9,10]. CAC can affect multiple organs, including the lungs, heart, brain, and kidneys, leading to their damage [11]. Subsequent studies showed that CAC differs from the classic disseminated intravascular coagulation observed in other infections [12,13]. However, numerous data indicate that, as in the cases of other coagulopathy-associated infectious diseases, the development of CAC is due to disturbances in the complex interactions between the plasma enzymes (coagulation, fibrinolytic, and kallikrein-kinin) and complement system and other elements of innate and adaptive immunity that determine the development of inflammation in response to infection [13,14,15]. These observations formed the basis of a concept called “immunothrombosis” [14,16,17,18]. This concept suggests that thrombosis is based on complex relationships between the vascular endothelium, other immune and non-immune cells, and multiple circulating and cell surface-associated factors that regulate coagulation processes and innate and adaptive immunity [19]. This concept has already been confirmed in studies of thrombosis development in cancer, sepsis, and ARDS [20,21,22]. However, the mechanisms of molecular and cellular interactions that determine the development of immunothrombosis in COVID-19 remain unclear.

When penetrating the body, the SARS-CoV-2 virus causes an immune response mediated by a few innate immune systems at once—pattern recognition receptors and the complement system [23,24,25,26]. Both systems cause the development of an inflammatory reaction aimed at limiting the spread of infection and destroying the pathogen. Numerous data indicate that severe COVID-19 is associated with disturbances in the regulation of the inflammatory response [27]. We believe that hyperinflammation in COVID-19 is caused not only by disturbances in the functioning of pattern recognition receptors and the complement system, but also by synergism in their work, which can enhance the response to infection. At the same time, they mobilize immune cells, involve the vascular endothelium in the pathogenetic process, and activate the blood coagulation system and platelets [28,29,30]. The coagulation system is closely linked to the innate immune system through multiple regulatory pathways, and its activation can lead to positive feedback loops that enhance the inflammatory response and worsen endothelial injury and hypercoagulability [31]. Ultimately, this leads to thrombotic complications, organ and tissue damage, and death. In this review, we discuss the features of immunothrombosis in COVID-19, as well as possible mechanisms of interaction between the immune system, the coagulation system, and the fibrinolytic system that may underlie endothelial injury and the development of a hypercoagulable state. Understanding the mechanisms of this disease will allow us to develop more effective treatments, save lives, and prevent complications.

## 2. Primary Immune Response to SARS-CoV-2 Infection

The penetration of a virus into the body causes the activation of a whole range of innate immune mechanisms; the actions of these mechanisms are aimed at the fastest possible recognition and destruction of a pathogen foreign to a given organism. The most important role in this primary response to infection is played by pattern recognition receptors (PRRs), primarily toll-like receptors (TLRs), and the complement system. In this section, we review the most recent evidence on how activation of TLR signaling and the complement system may occur during SARS-CoV-2 entry into the body, as well as potential interactions between these two systems and their role in the development of inflammation in response to infection.

### 2.1. TLR Family

Innate immunity is the body’s first line of defense against pathogen invasion. Innate immune cells recognize foreign and potentially dangerous biological agents using pattern recognition receptors (PRRs) present on cell membranes, in the cytosol, and in secreted form (soluble PRRs) [32]. Membrane PRRs include toll-like receptors (TLRs), which play a central role in activating innate immune cells and eliciting an immune response to infection and cell damage [33,34,35]. TLRs recognize a variety of pathogen-associated molecular patterns (PAMPs) and damage-associated molecular patterns (DAMPs) [36,37,38]. PAMPs are conserved molecular structures common to pathogens, including viruses, that enter the body [39,40]. In turn, DAMPs are usually endogenous molecules, the release of which signals cell damage and/or death [41]. Binding to the PAMP/DAMP pattern recognition receptor triggers a cascade of molecular interactions between factors of signaling pathways, which ultimately lead to the activation of proinflammatory gene expression and the initiation of innate immunity [36]. Thus, binding of various pathogen molecules to TLRs causes conformational changes in their extracellular part, which are transmitted to their intracellular TIR domain [36]. Further signal transmission can be mediated by adapter proteins MyD88, TRIF, TIRAP, TRAM, and SARM [42,43]. Each receptor of the TLR family has its own set of adapter proteins which it is able to bind and activate [42,43]. These adapter proteins mediate the activation of the synthesis and secretion of proinflammatory and anti-inflammatory cytokines [44]. Normally, activation of TLRs by molecules contained in SARS-CoV-2 viral particles should lead to activation of innate immunity and rapid elimination of the virus from the body [40,45,46]. However, if a viral infection causes hyperactivation of TLRs and disrupts their intracellular signaling, TLR-mediated inflammation may be excessive and cause damage to organs and tissues [47,48]. It has been shown that the development of severe forms of COVID-19 is associated with the excessive, uncontrolled release of cytokines, which can be caused by excessive activation of TLRs [49,50]. This fact served as the basis for the inclusion of TLRs in the list of potential targets for pharmacological therapy for COVID-19 and limited use of their agonists and antagonists in treatment [51]. In this review section, we present the most comprehensive data to date on the mechanisms of interaction between SARS-CoV-2 and TLRs and their role in the pathogenesis of COVID-19.

#### 2.1.1. Structure and Classification of TLRs

**Figure 1:**
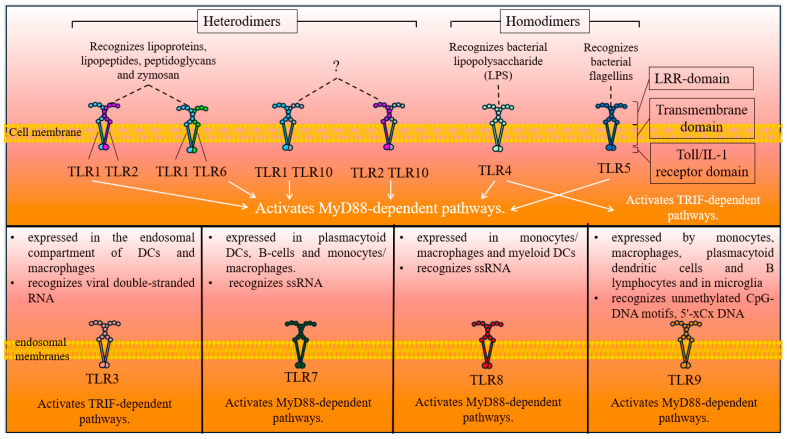
Structure and function of toll-like receptors (TLRs). TLRs are present on the cell surface and in the endosomes of many cell types. In resting cells, TLRs are present on the membrane in a monomeric state, but upon interaction with ligands they form dimers: homodimers (in most cases) or heterodimers (e.g., TLR2/TLR1). Binding of pathogen-associated molecular patterns (PAMPs) or patterns associated with damage to the body’s own cells (DAMPs) by the TLR receptor causes its dimerization and conformational changes in its structure. These conformational changes transmit a signal from the external domain responsible for ligand-binding to the internal TIR domain, activating it. For further signal transmission inside the cell, the TIR domain can recruit several adapter proteins, thereby activating two main signaling pathways: the MyD88-dependent pathway and the TRIF-dependent pathway. Ultimately, these signaling pathways trigger the expression of genes encoding proinflammatory cytokines, chemokines, and costimulatory molecules.

All TLRs have a similar structure and belong to the type I transmembrane glycoproteins [52]. The extracellular part of the receptor (N-terminus) is represented by the LRR domain (leucine-rich repeats), which is responsible for ligand binding (Figure 1) [52]. The single helical transmembrane domain, passing through the membrane into the cytosol, connects it with the internal part of the receptor—the C-terminal TIR domain (Toll/Il-1 receptor domain) (Figure 1). The TIR domain recruits adapter proteins and initiates signaling pathways that determine the effector function of the receptor [53]. When interacting with a ligand, TLRs form homodimers or heterodimers [53,54,55,56,57]. Dimerization is necessary for further signal transmission [53,54,55,56,57]. In this case, the cytoplasmic domains of TIR come together and form a platform for further signal transmission through adapter proteins and activation of the proinflammatory response [53,54,55,56,57]. 

TLRs are localized in the cell in such a way as to ensure recognition of molecular patterns of pathogens both in the extracellular space and inside the cell [43]. TLR1, TLR2, TLR4, TLR5, TLR6, and TLR10, integrated into the cytoplasmic membrane, recognize conserved molecular structures on the surfaces of pathogens upon their contact with the cell surface (Figure 1) [58].

Also, activation of TLRs on the cell surface can be mediated by intracellular and extracellular damage-associated molecular patterns (DAMPs) [59,60]. Intracellular DAMPs are molecules that are released from the body’s own cells during their activation and death [59,60]. Extracellular DAMPs include extracellular matrix molecules that are activated or degraded during tissue damage [59,60]. Integrated into the membranes of intracellular compartments (i.e., endosomes, lysosomes, and the endoplasmic reticulum), the TLR3, TLR7, TLR8, and TLR9 are responsible for recognizing pathogen-specific molecular patterns inside the cell (Figure 1) [58]. Primarily, the ligands of intracellular TLRs are pathogen nucleic acids, as well as nucleic acids of the body’s own cells released during cell death [61]. However, there is increasing evidence indicating the ability of intracellular TLRs to be activated by other endogenous patterns [62,63]. It is important to note that TLR3, TLR7, and TLR9 can be exposed on the cell surface and still maintain their functional activity [64]. The predominantly intracellular localization of TLRs that recognize nucleic acids prevents their activation by endogenous ligands and the development of autoimmune reactions [65]. Of all NA-recognizing TLRs, TLR3 is the most widely represented on the cell surface, and the endogenous ligands of which are least widespread in the extracellular environment [65]. The exposure of intracellular TLRs on the cell surface may be due to the need to enhance the immune response to infection [64]. In some cases, the activation of TLRs, in particular by endogenous molecules during cell and tissue damage, can only worsen the condition and lead to the development of chronic inflammation [59]. 

TLR2 is widely present in the body and is found in immune cells and endothelial and epithelial cells [66,67]. It has a wide range of recognized PAMPs, which include lipoproteins, lipopeptides, peptides, and zymosan (Figure 1) [68]. In addition to recognizing PAMPs, TLR2 plays an important role in the induction of a proinflammatory response to DAMPs. Human β-defensin-3, hyaluronan fragments, heat shock proteins, and high mobility group protein 1 can act as endogenous TLR2 ligands [69]. It has been reliably shown that after ligand binding for intracellular signal transduction, TLR2 forms heterodimers with TLR1 and/or TLR6 (Figure 1) [70]. There is also emerging evidence that TLR2 can form homodimers and heterodimers with TLR4 and TLR10, but the ligands that activate this dimerization and the functional roles of these dimers remain unclear [70]. There is evidence that suggests that the TLR2/TLR10 heterodimer mediates the proinflammatory response triggered by the binding of TLR2 to PAMPs of Listeria monocytogenes, Helicobacter pylori bacteria, influenza A virus, and molecular patterns associated with Parkinson’s disease [70]. 

TLR4 is mainly expressed by the cells of the immune system. Lipopolysaccharide (LPS) of the bacterial cell wall is considered the main ligand of the TLR4 (Figure 1) [71]. In addition to LPS, TLR4 is activated by a wide range of endogenous DAMPs, including high-mobility group box 1 (HMGB1), fibronectin EDA, fibrinogen, tenascin-C, surfactant proteins A and D, β-defensin-2, heat shock proteins, S100A8, S100A9, neutrophil elastase, antiphospholipid antibodies, lactoferrin, serum amyloid A, Oxydized low-density lipoprotein (Ox-LDL), saturated fatty acids, heparan sulfate fragments, and hyaluronic acid [59]. Recognition of the ligand by TLR4 causes its dimerization, which is necessary for triggering intracellular signal transduction and activating the expression of proinflammatory genes [72]. 

TLR5 is expressed in epithelial cells and immune cells such as monocytes and dendritic cells [73,74]. This is the only receptor in the TLR family that recognizes flagellins, the structural proteins of bacterial flagella (Figure 1) [75]. 

TLR3 have been identified in neurons, immune cells, fibroblasts, and various epithelial cells [76,77,78,79,80]. Among immune cells, only myeloid dendritic cells (DCs), macrophages, and mast cells (MCs) express TLR3 [81,82,83]. Double-stranded RNAs act as TLR3 ligands (Figure 1) [84]. These can be double-stranded genomic RNAs of viruses or intermediate products of viral replication whose genome is represented by a single-stranded RNA or DNA molecule [84]. TLR3 can also be activated by mRNA molecules, which can originate from damaged and dying cells [85]. The extracellular domain of the TLR3 contains two sites that are responsible for ligand binding and an interaction site that ensures the formation of a homodimer [86]. Homodimer formation is required for stable ligand binding and activation of intracellular signaling [86]. 

TLR7, TLR8, and TLR9 receptors are found in immune cells. TLR8 receptors are predominantly found in the intracellular compartments of monocytes/macrophages and myeloid DCs [87,88], while TLR7 is predominantly expressed in plasmacytoid DCs and, to some extent, in B cells and monocytes/macrophages [89]. TLR9 expression is highest in plasmacytoid DCs. In monocytes, TLR9 expression is low or absent, in contrast to TLR8 [88,90]. TLR7 and TLR8 receptors bind ssRNA and, by forming homodimers, activate MyD88-dependent signaling pathways (Figure 1) [39,91]. This signaling pathway mediates the activation of the expression of genes encoding type I interferons (IFN) and inflammatory cytokines, their synthesis and secretion [39,91]. In addition to ssRNA, endogenous antiphospholipid antibodies can act as ligands for these receptors [62,63]. Unlike TLR7 and TLR8, the TLR9 receptor recognizes DNA (Figure 1) [92]. In the structure of the extracellular domain of the TLR9 receptor, two sites are distinguished, one of which is responsible for binding unmethylated CpG DNA motifs characteristic of viral and bacterial DNA, and the second—DNA molecules that contain cytosine in the second position from the 5′-end (DNA 5′-xCx) [92]. These sites ensure binding of the TLR9 receptor to the ligand and its dimerization to activate intracellular signal transduction [92]. In addition to CpG DNA and 5′-xCx DNA, TLR9 activation can be induced by an endogenous patterns such as the chromatin complex with IgG [93].

#### 2.1.2. Recognition of SARS-CoV-2 Components by TLR Receptors

TLRs play a key role in the fight against viral infections [94]. The SARS-CoV-2 virus is no exception [23]. SARS-CoV-2 viral particles contain a wide range of molecules that can activate both surface and intracellular TLRs. First of all, these are structural proteins of the lipoprotein envelope, nucleocapsid proteins, and genomic single-stranded (+) RNA (+ssRNA) [23]. Viral proteins are recognized by cytoplasmic TLRs, which are widely represented on different types of cells, which leads to the initiation of a proinflammatory immune response [23]. A key role in the activation of TLRs integrated into the cytoplasmic membrane of cells is assigned to the structural proteins of the SARS-CoV-2 virus envelope, S (spike protein), M (membrane protein), and E (envelope protein), as well as the nucleocapsid protein N [23]. The S protein is the main structural protein of SARS-CoV-2, one which ensures interaction with angiotensin-converting enzyme II (ACE2) and other host cell receptors that mediate the penetration of the virus into the cell [95,96]. Structurally, the S protein is divided into a signal peptide located at the N-terminus, the S1 subunit, and the S2 subunit. The S1 subunit is responsible for binding to receptors, and the S2 subunit ensures the fusion of the viral lipoprotein envelope with the host cell membrane [97].

The S protein is recognized by TLR2 on the surfaces of macrophages, monocytes, and lung epithelial cells [98,99]. By binding the S protein, the TLR2 receptor forms heterodimers with TLR1 or TLR6 and, through the activation of the intracellular MyD88-mediated signaling pathway, triggers the expression and secretion of proinflammatory cytokines and chemokines [96]. In addition to the S protein, TLR2 is able to bind the E and N proteins of the SARS-CoV-2 virus, but only the interaction of the N protein with the TLR2 receptor can cause its activation [100,101]. SARS-CoV-2 viral particles do not merely activate the TLR2 receptor. It has been shown that SARS-CoV-2 viral infection stimulates the expression of the TLR2, TLR7, IRF3, CD36, MDA5, and ACE2 genes in cells, thereby enhancing the proinflammatory response [102,103]. At the same time, a correlation was found between the level of TLR2 expression and the severity of the disease, which emphasizes the important role of TLR2 in the pathogenesis of COVID-19 [101]. In addition to TLR2, structural proteins of the SARS-CoV-2 virus envelope can activate the TLR4 receptor. The ability of TLR4 to bind the S spike protein was demonstrated by molecular docking and surface plasmon resonance (SPR) methods [104,105]. It has been shown that activation of the proinflammatory response by the S protein S1 subunit in microglia, macrophages, and TLR4-signaling transgenic HEK293 cells is mediated by the MyD88 factor and can be suppressed by specific TLR4 inhibitors [104,106,107]. 

Contacts of viral particles with cells do not merely induce a TLR-mediated proinflammatory response. Viral particles interact with other receptors that ensure their penetration into the cell. One such receptor is angiotensin-preferring enzyme 2 (ACE2) [108]. It binds the structural protein S of the SARS-CoV-2 virus envelope and ensures viral penetration into the cell via two different pathways: clathrin-dependent endocytosis and fusion of the viral membrane with the cell cytoplasmic membrane [108]. In the latter case, additional participation of transmembrane serine proteinase 2 is required, which, after binding of ACE2 to the S protein of the virus, cleaves the S protein at the S1/S2 and S2 sites, thereby ensuring the fusion of the viral membrane with the host cell membrane and penetration of the contents of the viral particle into the cytoplasm [108]. In both cases, the molecular patterns of the SARS-CoV-2 virus become accessible for recognition by intracellular TLR receptors. Single-stranded RNA of the SARS-CoV-2 virus genome and an intermediate product of its replication in the form of double-stranded RNA act as ligands that activate intracellular TLR receptors. Using a model of multicellular spheroids, it was demonstrated that infection with the SARS-CoV-2 virus causes activation of RNA-recognizing receptors TLR3 and TLR7/8 [45]. In this case, TLR7/8 receptors trigger a proinflammatory response via the IRF-7 and MyD88-NF-κB signaling pathways, thereby causing increases in the production of type I IFN and proinflammatory cytokines [91]. In turn, TLR3 activates the TRIF-mediated signaling pathway, but not MyD88 [44]. TRIF recruits TRAF6 and TRAF3 proteins, triggering a chain of molecular interactions that lead to the activation of the transcription factor IRF3 [44]. The latter penetrates the nucleus and triggers the expression of IFN-α and IFN-β [44]. IFN-α and -β contribute to the elimination of invading viruses, causing the death of infected cells, imparting resistance to viral infection to surrounding cells and activating protective reactions of the adaptive immune system [44,109]. Thus, TLRs are involved in the fight against viral infection at all its stages, modulating the immune response. 

Other PRRs, such as retinoic acid-inducible gene I (RIG-I) and melanoma differentiation-associated protein 5 (MDA5), known as RIG-I-like receptors (RLRs), are also involved in the recognition of viral RNAs [110]. Signaling from RIG-I and MDA5 leads to the activation of the transcription factors IRF3, IRF7, and NF-kB, which in turn trigger the expression of interferon genes and proinflammatory cytokines, such as TNF-α, IL-6, and IL-1β [110]. Autoamplification promotes the expression of each [111].

#### 2.1.3. The Role of Toll-like Receptors in the Development of Acute Inflammatory Response in COVID-19

Activation of TLRs plays a dual role in the progression of COVID-19. On the one hand, TLR recognition of the molecular patterns of the SARS-CoV-2 virus and the immune response they trigger are necessary for the body to fight the infection. At the same time, the IFN signaling cascade plays a key role in the TLR-mediated response to SARS-CoV-2 infection [112]. A number of facts can serve as indirect confirmation of the important role of these receptors in the fight against COVID-19 infection. It is known that infection with the SARS-CoV-2 virus is accompanied by an increase in the expression of TLR3 and TLR7 [91]. TLR3-/- mice are more susceptible to coronavirus infection and demonstrate increased mortality compared to the wild type [45,113]. Deficiency of TLR7 functional activity is associated with a strong predisposition to severe COVID-19 in men [114]. Finally, the TLR7 and TLR8 genes are located on the X chromosome. Women have a double set of alleles compared to men. Biallelic expression of the TLR7/8 genes and, as a result, their increased activity in women may be one of the reasons for their relatively high resistance to infection [115]. However, the SARS-CoV-2 virus has a whole set of tools to suppress the INF response [112]. Thus, it was shown that the nsp and ORF proteins, structural proteins M and N, and papain-like protease PLpro, encoded by the genomic RNA of the SARS-CoV-2 virus, are able to suppress IFN production and block the INF-activated JAK-STAT signaling pathway, and, as a consequence, STAT-mediated expression of interferon-stimulated genes (ISG) [112]. 

On the other hand, activation of TLRs such as TLR2, TLR4, and TLR7/8 leads to the release of proinflammatory cytokines by cells, a phenomenon which studies have shown to largely determine the development of complications in COVID-19 [116]. Increased levels of proinflammatory cytokines, the expression of which is activated by TLR4 signaling pathways, are observed in COVID-19 patients [117]. Other studies have found a link between disease severity and TLR7 receptor activation [114,118]. Neutrophil extracellular traps (NETs), which are actively formed in severe COVID-19 patients, can also activate various pattern recognition receptors, including TLR4 and TLR7, thereby facilitating the release of inflammatory mediators [119]. Increased expression of TLRs in obese individuals also correlates with significantly elevated levels of proinflammatory cytokines IL-6 and TNF-α, and more severe disease [118,120]. These findings highlight the clinical importance of the TLR-mediated immune response to infection. 

In addition to pathogen-associated molecular patterns, endogenous molecular patterns released during cell damage and death (DAMPs) may be involved in TLR activation and the development of an inflammatory response. Viral replication leads to pyroptosis, a highly inflammatory form of lytic-programmed cell death (apoptosis) [121]. In patients with COVID-19, pyroptosis causes activation of proinflammatory cytokine secretion and changes in the functional activity of macrophages and lymphocytes [121,122], which are manifested by the development of peripheral lymphopenia [123]. This proinflammatory response may be caused by activation of TLR receptors by DAMPs released during pyroptosis. One such DAMP may be HMGB1. It has been shown that in COVID-19 patients, elevated HMGB1 levels positively correlated with proinflammatory cytokine levels and disease severity [124]. As a TLR4 ligand, HMGB1 can trigger a TLR4-mediated proinflammatory response, which is expressed in the activation of IL-1β, IL-6, and TNF-α production [124,125,126]. Another DAMP may be calprotectin (S100A8/A9) [127]. It is released from host neutrophils and, as researchers suggest, is a biomarker of particularly severe forms of COVID-19 with a fatal outcome [127]. Mitochondrial damage and cell death in COVID-19 also result in increased levels of extracellular DNA (cfDNA) [128,129]. CfDNA as a ligand for TLR9 can also enhance the proinflammatory response [130]. CfDNA in COVID-19 patients has been shown to induce TLR9-mediated mitochondrial ROS (mtROS) production, and elevated cfDNA levels correlate with disease severity [131,132]. Thus, in addition to viral PAMPs, DAMPs released as a result of SARS-CoV2-induced cell death may also be involved in TLR activation and inflammation in COVID-19. The data also suggest that the uncontrolled inflammatory response in COVID-19 may be based on excessive activation of TLR-mediated expression of proinflammatory cytokines and disturbances in the TLR-mediated interferon response to viral infection.

### 2.2. Complement System

The complement system is one of the humoral factors of innate immunity. It is a system of more than 50 proteins that perform their functions in blood plasma, both on the surface and inside of the cells. These include the main proteolytic components of the complement system, various regulatory proteins, cofactors, anaphylatoxins, and receptors, which together provide immune surveillance and tissue homeostasis [133,134]. Complement is involved in the recruitment and priming of leukocytes, inflammatory processes, and vascular reactions; facilitates phagocytosis; and causes direct lysis of pathogens due to the formation of a membrane attack complex [135,136]. In this case, complement can direct its effector functions not only to foreign agents, but also to various altered structures of the body itself [134]. It has been shown that complement, being part of innate immunity, is nevertheless a modulator of adaptive immune reactions [137]. Thus, it has been established that C3a and C5a enhance the proliferation and differentiation of naive T cells and promote their survival, and that C3dg, a C3b cleavage product, through binding to the CR2/CD19 co-receptor complex lowers the threshold for B-cell activation and promotes their maturation [138,139,140]. Recent studies indicate the roles of the intracellular proteins of the complement system in maintaining normal cell physiology, in particular participation in biochemical reactions of glycolysis and oxidative phosphorylation, ensuring cell survival and regulating gene expression [141]. The functions of complement also include participation in the clearance of immune complexes, angiogenesis, mobilization of hematopoietic stem cells/progenitor cells, and tissue regeneration, as well as many others [140]. However, in this review, we will consider the main function of complement as a humoral factor of innate immunity—protection—and complement’s relationship with the blood coagulation system, as well as how hyperactivation of complement can lead to the complications of COVID-19.

#### 2.2.1. Complement Activation Pathways

**Figure 2:**
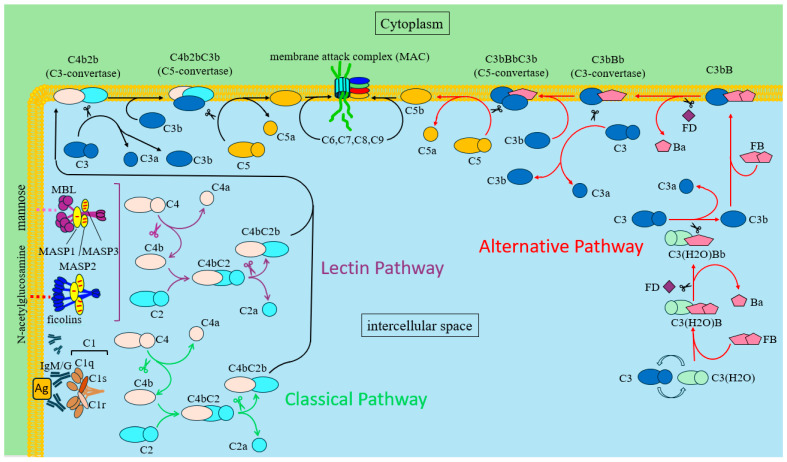
Scheme of complement-activation pathways. Activation of the classical pathway is initiated by binding of the antigen–antibody complex to factor C1q, which is part of the initiating complex C1. Complex C1 also contains 4 zymogens: 2 molecules of C1r and 2 molecules of C1s. Binding of the antigen–antibody complex to factor C1q causes autoactivation of C1r proteases. They then activate C1s proteases. C1s proteases sequentially cleave C4 to form fragments C4b and C4a, and C2 as part of a complex with C4b. As a result, the complex C4b2b—C3 convertase CP/LP is formed. The lectin pathway is activated by the complex of the pattern recognition receptor MBL or FCN with zymogens MASP-1, MASP-2, and MASP-3. By binding to polysaccharides on the pathogen surface, MBL/FCN activates the MASP-1 zymogen. In turn, the activated MASP-1 protease cleaves the MASP-2 zymogen. The activated MASP-2 protease cleaves C4 and C2, thereby mediating the formation of the C3 convertase CP/LP. The alternative activation pathway is based on spontaneous hydrolysis of C3, which results in the formation of the active form C3(H_2_O). C3(H_2_O) binds factor B, ensuring its cleavage by protease D. As a result, the initiating C3 convertase of the alternative pathway C3(H_2_O)Bb is formed. It cleaves C3 to form fragments C3b and C3a. C3b also binds factor B, ensuring its cleavage by protease D. As a result, the C3 convertase of the alternative pathway C3bBb is formed. C3 convertases C4b2b and C3bBb cleave C3 to form C3b and C3a fragments. In turn, the C3b fragment forms complexes with C3 convertases C4bC2bC3b and C3bBbC3b. These complexes are able to cleave factor C5 to form fragments C5b and C5a. C5b sequentially recruits complement factors C6-C9, forming a lytic membrane attack complex—a pore in the cytoplasmic membrane of the target cell. Anaphylatoxins C3a and C5a, small cleavage fragments of C3 and C5, respectively, do not participate in the formation of the lytic complex, but affect many immune/non-immune cells by mediating chemotaxis and inflammation and performing a number of other functions.

There are three main pathways of complement activation: classical (CP), lectin (LP), and alternative (AP) (Figure 2) [142,143]. All three main activation pathways represent a cascade of sequential interactions between complement system factors, one which ultimately leads to the formation of a membrane attack complex, a transmembrane pore that causes lysis and cell death [142,143]. Complement system factors that are involved in the classical pathway and partially in the lectin/alternative pathway are traditionally designated by the letter C [142,143]. Factors involved in the activation of the lectin and alternative pathways have a different designation [142,143]. During activation, some complement proteins undergo cleavage into two unequal fragments which are designated by adding the letter “a” for a small fragment and “b” for a large one [142,143]. The complement-activation pathways differ in their initiation mechanisms [142,143,144,145,146,147,148,149]. However, the initiation of each of them causes the sequential formation of C3 and C5 convertases, and the terminal stage of MAC formation is common to all three pathways (Figure 2) [142,150].

The classical pathway was originally thought to be initiated by the recognition of an IgM or IgG complex with an antigen by complement factor C1q (Figure 2) [136,143]. Subsequent studies have shown that activation of the classical pathway can be induced by the binding of factor C1q to other ligands, such as C-reactive protein and other pentaxins, extracellular matrix proteins, amyloid deposits, DNA molecules, and prions [143,151,152]. Complement factor C1q is part of the C1 complex [153,154]. In addition to C1q, the C1 complex contains two C1r molecules and two C1s molecules [153,154]. C1r and C1s are protease zymogens [153,154]. Binding of the antigen–antibody complex by factor C1q causes its conformational changes, which presumably result in autoactivation of C1r [153,154]. Activated C1r proteases in turn activate C1s proteases by cleaving zymogens [153,154]. After activation, C1s proteases in the C1 complex acquire the ability to cleave factor C4 and factor C2 in the C4bC2 complex, which ultimately leads to the formation of C3 convertase C4b2b and anaphylatoxins C4a and C2a (Figure 2) [155,156]. 

The lectin pathway is initiated by mannose-binding lectin (MBL) and ficolins (Figure 2) [147,157]. MBL and ficolins are pattern recognition receptors and contain domains that recognize carbohydrate structures, primarily N-acetylglucosamine and mannose, on the surfaces of various pathogens [157,158,159]. Similar to the C1q receptor of the classical pathway, MBL and ficolins form a complex with protease zymogens that are sequentially activated upon binding of MBL and ficolins to a ligand [157,160]. These proteases are called MBL-associated serine proteases (MASPs). MASP-1 is similar to the C1r protease and performs a similar function by activating the downstream protease MASP-2 [157]. Like C1s, MASP-2 cleaves C4 and C2 to form the C3 convertase C4bC2b, thereby activating the complement system (Figure 2) [161]. 

The alternative complement pathway differs fundamentally from other pathways in that it does not require recognition of any molecular patterns for its activation [162]. This pathway is capable of self-activation and normally exists in a state of permanent low activity [162]. This ability of the alternative pathway is due to the constant spontaneous hydrolysis of the thioether bond within the complement component C3, which leads to the formation of the active form C3(H_2_O) (Figure 2) [162,163]. Normally, the formation of C3(H_2_O) occurs at a low rate, but this process can be significantly accelerated by chaotropic or nucleophilic agents or by contact of C3 with various surfaces [164]. Enhancement of C3(H_2_O) generation and activation of the alternative pathway have been demonstrated upon contact with glass, polystyrene, bare polypropylene, and polypropylene coated with bacterial LPS [164]. C3(H_2_O) has the ability to form a complex with factor B similar to C3b [164,165]. Binding of C3(H_2_O) to factor B causes conformational changes in the latter, which make it accessible for cleavage by a serine protease called factor D (Figure 2) [165]. Factor D cleaves factor B into two unequal fragments, the larger of which (Bb) remains in the C3(H_2_O)Bb complex (Figure 2) [163,165]. The small fragment is called Ba. C3(H_2_O)Bb has protease activity and is called the initial liquid-phase C3 convertase of the alternative pathway [165]. The key function of this complex is to cleave C3 to form C3b and C3a fragments, which ultimately leads to the formation of another C3 convertase of the alternative pathway (Figure 2) [165]. Thus, some of the C3b molecules form a complex with factor B, after which it is cleaved by factor D to form Ba and C3bBb, the alternative pathway convertase (Figure 2) [166,167,168]. Fragment C3a does not participate in the further process of complement activation. It is an anaphylatoxin that has multiple activities; in particular, it mediates the development of an inflammatory reaction (Figure 2) [169].

C3 convertases of all three complement-activation pathways cleave C3 to C3a and C3b (Figure 2) [170,171]. In this process, some of the C3b molecules are recruited to form new C3 convertase complexes of the alternative pathway [172]. This is the mechanism by which the alternative complement pathway can amplify the overall activity of the complement system, regardless of the pathway by which it was activated [172]. Also, the C3b fragment, forming covalent bonds with molecules on the surface of the pathogen, ensures their recognition and phagocytosis [173]. Finally, C3b binds to C3 convertases, forming complexes C4b2bC3b and C3bBbC3b (Figure 2) [174,175]. These complexes are called C5 convertases of the classical/lectin (C4bC2bC3b) and alternative (C3bBbC3b) pathways [175,176]. Unbound C3b is hydrolyzed [161]. C5 convertases of all three complement-activation pathways have the same functional activity [175,176]. They cleave factor C5 to form two fragments: a large fragment of C5b, which participates in the assembly of MAC, and anaphylatoxin C5a, which has pronounced chemotactic and anaphylatoxic activity (Figure 2) [169,175,176]. Membrane-bound C5b sequentially recruits complement components C6, C7, and C8 [177]. Inclusion of factors C7 and C8 in the forming complex ensures its integration into the cytoplasmic membrane of the target cell (Figure 2) [178,179]. Formation of the membrane attack complex is completed by the inclusion of 12–20 molecules of C9, which form a membrane-penetrating pore [177,179]. It is important to note that the functions of MAC are not limited to cell lysis. MAC stimulates the polarization of T-helper cells and participates in platelet activation [150,180]. 

Data obtained in recent years have shown that the mechanisms of complement activation are not limited to the three pathways described above. Complement can be activated through mechanisms that do not require the participation of complement-initiating complexes or the hydrolysis of C3. For example, the coagulation protease thrombin can cleave C5 with subsequent formation of MAC [181].

#### 2.2.2. Regulation of the Complement System

The complement system not only ensures a rapid response to the penetration of pathogens into the body, but also participates in maintaining the homeostasis of organs and tissues by mediating the removal of the body’s own altered, damaged, or dying cells [140]. In this case, the complement system is in a constant state of low activity and is capable of rapidly increasingit, as mentioned above [162]. In this situation, fine regulation of the complement system cascades is urgently needed to protect normal cells from complement-mediated damage [162]. Disturbances in complement regulation lead to the development of autoimmune diseases and damage to organs and tissues [182]. Regulation of the complement system is provided by a whole complex of complement system inhibitors circulating in the blood plasma, associated with the cell surface, and integrated into the cell membrane [183]. These factors ensure the regulation of the complement system activity at all stages, from initiation to the formation of the lytic complex [182]. Initiation of the classical and lectin pathways of complement activation is under the control of the C1 esterase inhibitor (C1-INH). This is a secreted glycoprotein that inhibits the activity of serine proteases C1r and C1s as part of the C1 complex of the classical pathway. It also suppresses the activity of proteases MASP-1 and MASP-2, which, in complex with MBL or ficolins, activate the lectin pathway [184]. The activity of most complement regulators is aimed at limiting the formation and inactivation of C3 convertases. These include decay accelerating factor (DAF; CD55), complement receptor type 1 (CR1, CD35), membrane cofactor protein (MCP; CD46), C4b binding protein (C4BP), factor H, and factor I [183]. DAF is a transmembrane glycoprotein that is present on the surface of almost all nucleated cells [185]. Its extracellular portion contains four domains that are responsible for complement control. DAF inhibits autologous complement activity by accelerating the dissociation of cell surface-bound C3 convertases of all three complement-activation pathways [185]. The integral membrane proteins MCP and CR1 bind C3b and C4b and function as a cofactor in factor I-mediated cleavage of C3b and C4b [186,187,188]. In addition to its cofactor function, CR1 accelerates the degradation of C3 and C5 convertases of all three complement-activation pathways by competitively displacing factors Bb and C2b from these complexes [189,190]. In the fluid phase, C4BP and factor H function as factor I cofactors [191]. Factor H has also been shown to block the binding of C3b to factor B [192]. Finally, in addition to its activity in blood plasma, factor H prevents surface activation of the alternative pathway by binding to heparan sulfates and glycosaminoglycans on the cell surface [193,194]. Factors H and MCP are considered to be regulators of the alternative pathway, while C4Bp and DAF are considered regulators of the classical and lectin complement-activation pathways. Finally, there are factors that inhibit complement at the stage of MAC formation. These include protectin (CD59), vitronectin, and clusterin [195,196,197]. Thus, the cellular regulator CD59 prevents the formation of both sublytic and lytic complexes [140,197,198].

#### 2.2.3. Activation of the Complement System in COVID-19

The complement system plays a key role in the pathogenesis of infection caused by SARS-CoV-2 [24,25,26]. It is one of the most important elements of innate and adaptive immunity, and has a wide range of functions [140]. The complement system is constantly active and, in a normal state, controls homeostasis, ensuring the rapid elimination of damaged or dead cells, their fragments, and other cellular debris [140,162]. It is the first entity to engage in the fight against the infectious agent when the latter enters the body [140]. However, hyperactivation of the complement system, as well as incorrect recognition of cellular debris, can lead to various pathological conditions, such as the development of thrombotic microangiopathies and MOF [199,200]. 

**Figure 3:**
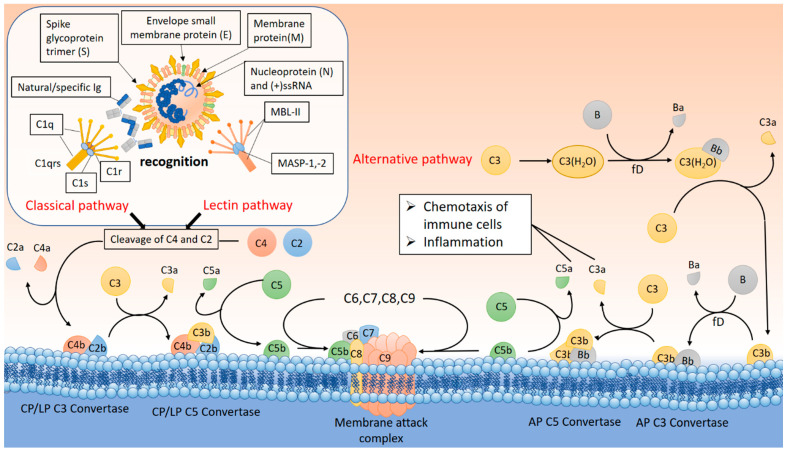
Pathways of complement activation by the SARS-CoV-2 virus. The SARS-CoV-2 virus activates all three canonical complement pathways. At the very onset of infection, activation of the classical complement pathway can be mediated by natural antibodies to evolutionarily conserved epitopes and is enhanced by the appearance of antibodies specific to viral proteins. One of the mechanisms of lectin pathway activation may be direct potentiation of MASP-2 proteinase by the SARS-CoV2 N protein. The alternative complement pathway is activated by binding of protein S to heparan sulfate on the cell surface, which is thought to disrupt factor H function.

Numerous data indicate that activation of the complement system occurs at the very onset of SARS-CoV-2 infection [201,202]. It has been shown that the development of COVID-19 is accompanied by an increase in the complement-activation products C3a and MAC, as well as C3c, a product of C3b proteolysis, in the blood plasma of patients [201]. Moreover, the plasma levels of C3a and MAC correlated with the severity of the disease, while the level of C3c did not differ between ICU patients and patients with a less severe course of COVID-19 [201]. In another study, activation of the complement system was observed in COVID-19 patients, which was reflected in increased content levels of C4d, C3bBbP, C3bc, C5a, and sC5b-9 in the blood plasma [202]. These data indicate that SARS-CoV-2 infection triggers several pathways of complement activation (Figure 3). For example, an increase in the level of C3bBbP indicates the activation of the alternative pathway. Activation of the alternative pathway may be mediated by the interaction of SARS-CoV-2 viral particles with heparan sulfate on the cell surface [203]. In this case, the structural protein S of the SARS-CoV-2 viral envelope may play a key role in the activation of the alternative pathway [204].

Elevated C4d levels indicate activation of the classical and/or lectin pathways. At the same time, the concentration of sC5b-9, as the end-product of activation of all three complement pathways, correlated most with respiratory failure and systemic inflammation [202]. The assumption that the lectin pathway of the complement system is activated in COVID-19 is supported by the data obtained by Gao et al. [205]. They demonstrated that dimers of the N protein of MERS-CoV, SARS-CoV-1, or SARS-CoV-2 viruses activate mannan-binding lectin-associated serine protease 2 (MASP-2), the main inducer of the lectin pathway (Figure 3) [205]. Moreover, deletion of Masp-2, or blocking the interaction of the N protein with MASP-2, attenuated lung injury [205]. Activation of the classical complement pathway at the onset of SARS-CoV-2 infection can be mediated by natural antibodies having specificity for evolutionarily conserved epitopes. These antibodies circulate in the blood, providing an immediate response to the infection (Figure 3) [206]. Indirect confirmation of the involvement of natural antibodies in the development of COVID-19 can be found in the fact that the level of infection and mortality from COVID-19 is lowest in people with blood group O [207,208,209]. In the context of the measles virus, it was shown that the blood group determines the glycosylation of viral particles with carbohydrate epitopes corresponding to the blood group [210]. At the same time, human preimmune serum containing natural neutralizing antibodies partially neutralized the virus with carbohydrate epitopes of type A and type B, but not type O, in a complement-dependent manner [210]. Since type O patients contain natural antibodies against both A-type and B-type carbohydrate epitopes, these antibodies may help reduce the viral load in their hosts through early activation of the classical complement pathway and low viral clearance before the development of pneumonia. Furthermore, coexpression of the SARS-CoV-1 spike protein (S) ectodomain with alpha1,2-fucosyltransferase and A-transferase in CHO cells was shown to impair the adhesion of these cells to ACE2-expressing cell lines in the presence of both S antibodies and human natural IgM against the A antigen [211]. Taken together, these data indicate that natural antibodies to blood group antigens may serve as an initial barrier to SARS-CoV-2 viral infection by triggering the classical complement pathway. Thus, activation of the complement system in COVID-19 can be mediated by three pathways at once. At the same time, the ability of the alternative pathway to amplify the complement-mediated response triggered by other activation pathways can play a negative role in the development of COVID-19. It has been established that activation of the alternative complement pathway is most pronounced in patients with severe disease requiring mechanical ventilation, correlates with markers of endothelial activation and damage, and is a potential predictor of an unfavorable outcome [212,213,214]. In this case, the activity of the alternative pathway can reach 80-90% of the total activity of the complement system [172].

### 2.3. Interactions Between TLRs and the Complement System as a Factor Amplifying the Inflammatory Response in COVID-19

#### 2.3.1. Synergistic Interactions Between TLRs and the Complement System in Response to Infections

Above, we presented data showing that two systems, the pattern recognition receptor system (mainly TLRs) and the complement system, are involved in the development of the cytokine storm-mediated excessive inflammatory response in COVID-19 patients. Numerous data indicate that both of these systems closely interact with each other, providing the primary response to infection [215]. These interactions apparently play a regulatory role. Synergism in the work of these systems may be the cause of the excessive inflammatory response or a factor that further enhances it [215]. Both systems are involved in the primary response to infection and are able to recognize the same molecular patterns. Thus, LPS is not only a natural ligand of TLR4, but also a well-known activator of the complement system [216,217]. In turn, zymosan, being a ligand of TLR2/6, can also activate the alternative pathway of the complement system [218]. Synergism between TLRs and the complement system has been demonstrated in the DAF-/- mouse model [219]. Compared to wild-type mice, LPS administration to DAF-/- mice caused a significantly greater increase in the levels of proinflammatory cytokines IL-6, TNF-α, and IL-1β and a decrease in IL-12 levels [219]. Subsequent analysis showed that the differences in the proinflammatory response to LPS between DAF-/- mice and wild-type mice were due to higher levels of expression of genes encoding proinflammatory cytokines in DAF-/- mice [219]. This fact formed the basis for the assumption about the involvement of the complement system in the regulation of the expression of proinflammatory cytokines. The authors demonstrated that complement activation by the cobra venom factor CVF itself does not cause changes in the level of IL-6, TNF-α and IL-1β, but enhances the expression and secretion of proinflammatory cytokines when LPS is administered to mice [219]. A more detailed study of this phenomenon showed that the complement system moderates the activity of TLRs through the C5a/C5aR and C3a/C3aR signaling pathways [219]. It synergistically enhances TLR4- and TLR2/6-induced production of proinflammatory cytokines IL-6, TNF-α, and IL-1β, but does not affect the production of these proinflammatory cytokines in cells activated by TLR9 ligand—CpG [219]. At the same time, the complement system suppresses the production of IL-12 by cells. This effect may be due to increased expression of IL-10 [219]. At least in part, the synergism between complement and TLRs depends on the cell type exposed and the complement factors involved. Binding of C3b by the complement inhibitor MCP and activation of the complement receptor CR3 by iC3b, as well as the gC1qR, C5aR, and C3aR signaling pathways, suppress IL-12 production by macrophages and/or monocytes [219,220,221,222,223,224]. In the case of LPS-stimulated dendritic cells, TLR-induced expression of IL-12 family cytokines can be stimulated by anaphylatoxins C5a and C3a, but is also suppressed by gC1qR activation [139,225,226,227,228]. The profile of cytokines produced by APCs in response to TLR activation determines the direction of T-cell differentiation [229,230,231]. It is known that the key role in the regulation of the Treg/Th-17 balance belongs to the proinflammatory cytokine IL-6 [232]. Thus, it was shown that the decisive role in the induction of Th17 differentiation in mice administered LPS is played by TLR-4 signaling-mediated secretion of IL-6 [233]. In addition to IL-6, cytokines of the IL-12 family (IL-12, IL-23, and IL-27) play an important role in the activation and differentiation of various T-cell subpopulations [234]. IL-12, indirectly, through the activation of IFNγ secretion, suppresses the development of IL-17-secreting cells, thereby limiting inflammation [235]. IL-23, on the contrary, stimulates the differentiation of IL-17-secreting T-cells [236]. IL-27 stimulates differentiation of naive CD4+ T cells into Th-1 and suppresses IL-17 secretion by activated CD+ T cells [237]. It is logical to assume that the nature of synergistic interactions between the complement system and TLRs may affect T-cell differentiation and associated effector functions. C5aR- and C3aR-mediated effects of anaphylatoxins on LPS-stimulated DCs stimulate differentiation of naive CD4+ T cells into Th-1 [139,225,226,227]. Conversely, combined activation of the TLR and C5aR signaling pathways in LPS-stimulated macrophages enhanced the pathways’ activity aimed at stimulating differentiation of CD4+ T cells into Th-17 [233]. It has been shown that synergism between the complement and TLR systems mediate enhancement of IL-6 production, promoted Th-17 differentiation in vivo and may cause the development of autoimmune encephalomyelitis or autoimmune arthritis [233,238].

It should be noted that the interaction between complement and TLRs is bidirectional. TLRs can also modulate the activity of the complement system, increasing the synthesis of complement proteins and its effector function [215]. Activation of TLR4 by LPS dramatically increases the transcription of the gene encoding factor B and its synthesis in macrophages [239]. TLR signaling pathways also affect cell sensitivity to C5a in vitro and in vivo. On the one hand, the authors showed that preliminary activation of toll-like receptors of blood mononuclear cells leads to an increase in the proinflammatory response to the effects of C5 [240]. On the other hand, it was shown that activation of peripheral blood mononuclear cells by LPS causes suppression of the expression of C5aR and C5L2 receptors [240] Thus, the interaction of complement and TLRs can not only enhance the innate immune response of the host, but also affect the factors of adaptive immunity.

#### 2.3.2. Potential Synergistic Interactions Between TLRs and the Complement System in COVID-19

**Figure 4:**
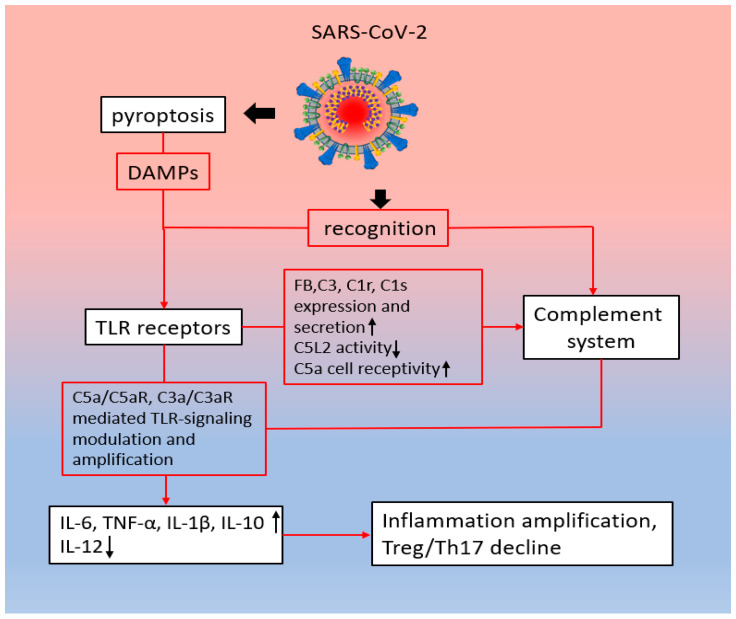
Synergy between TLR signaling and the complement system as a potential factor for amplification of the inflammatory response in COVID-19. The SARS-CoV-2 virus activates TLRs and the complement system. Activation of toll-like receptors can occur both directly upon recognition of viral PAMPs and through recognition of DAMPs formed during virus-induced cell pyroptosis. Activation of TLR signaling pathways can cause increased activity of the complement system by activating the expression and secretion of complement factors B, C3, C1r, and C1s and increasing the sensitivity of cells to anaphylatoxin C5a. In turn, the complement system can enhance the TLR-mediated response through the C5a/C5aR and C3a/C3aR signaling pathways. Thus, interactions between TLRs and the complement system may enhance TLR signaling-mediated release of proinflammatory cytokines. This leads to an increase in the inflammatory response and a shift in the balance of naïve T cell differentiation in favor of Th-17 (↓—downregulation, ↑—upregulation).

The role of interactions between the complement system and TLRs in the development of an uncontrolled inflammatory response in COVID-19 remains to be established. However, there is already evidence that this synergism in the work of the systems may be involved in the pathogenesis of COVID-19 (Figure 4). It was mentioned above that TLRs actively perceive spike proteins (N, S, or G) or mRNA of the NSP-10, S2, and E proteins of SARS-CoV-2 [118,241]. COVID-19 patients are characterized by increased activation of the complement system, which correlates with the severity of the disease [214]. Given the numerous interactions between complement and TLRs, it is logical to assume that they can jointly contribute to the modulation of the body’s proinflammatory response to COVID-19 infection. Elevated levels of IL-10 are observed in severely ill patients [242]. In these cases, blood plasma level of IL-10 is considered a predictor of disease severity [242]. Also, decreased IL-12 levels have been observed as being associated with a more severe course of the disease [243]. These changes in cytokine levels may be due to the synergistic effects of complement system components and TLRs on macrophages and monocytes. In turn, hyperactivation of macrophages and their increased infiltration into the lung tissue contribute to the development of acute respiratory distress syndrome, respiratory failure, and death in patients with COVID-19 [244,245]. It has also been established that the interaction of various PAMPs of the SARS-CoV-2 virus with TLRs of macrophages contributes to the polarization of macrophages according to the M1 phenotype (Figure 4) [241,246,247].

TLR or/and C5aR-mediated induction of IL-6 production and secretion by macrophages can lead to a shift in the balance in the differentiation of naive T cells towards Th-17 in COVID-19 patients. It is known that patients with COVID-19 have elevated levels of IL-6 (Figure 4) [248,249,250]. An elevated IL-6 level correlates with the duration of the disease [250] and is considered a predictor of disease severity and mortality [251]. High levels of IL-6 in patients with COVID-19 may be associated with an imbalance in the differentiation of naive T cells. Th17 cells produce IL-17A, IL-17F, IL-21, and IL-22 and are thought to play a crucial role in autoimmune inflammation [252]. Treg cells, on the contrary, express anti-inflammatory mediators (IL-4, IL-10, and TGF-β), thereby limiting the development of an inflammatory response [253]. In patients with severe COVID-19, there is a decrease in the number of Treg cells, which leads to an imbalance between Treg and Th17 cells in favor of Th17 [254,255]. The balance between Treg and Th17 cells determines the severity of uncontrolled systemic inflammation in acute lung injury (ALI) and acute respiratory distress syndrome (ARDS) [256,257]. Based on these data, it can be concluded that disturbances in the regulation of polarization of naive CD4+ T cells can contribute to the development of a hyperinflammatory response and tissue damage in patients with COVID-19 [253]. This can be confirmed by the fact that the development of severe forms of COVID-19 is associated with an increase in the population of Th17 cells in bronchoalveolar lavage fluid and an increase in the levels of proinflammatory cytokines IL-17 and GM-CSF produced by these cells and found in the blood and tears of patients [258,259,260,261,262,263,264,265,266]. There is evidence that in COVID-19, it is not only the case that the complement system can modulate the TLR-mediated response, but TLR signaling can also influence the activity of the complement system. SARS-CoV-2 viral particles have been shown to alter the expression profile of complement system genes in epithelial cells, activating the expression of factor B, C3, C1r, and C1s genes [267]. Studies of the mechanisms of this induction have shown that the activation of C3 and factor B expression is mediated by the type I interferon signaling pathway, the expression of which is activated by TLRs (Figure 4) [267]. Thus, at least part of the activation of complement system gene expression in COVID-19 may be mediated by TLRs.

### 2.4. Cytokine Storm in COVID-19

It has been established that the pathogenesis of COVID-19 is based on a systemic inflammatory response which is characterized by a pronounced release of proinflammatory cytokines, commonly known as a cytokine storm or cytokine release syndrome [268]. Proinflammatory cytokines are involved in a cascade of lung inflammation, hypercoagulation, and thrombosis, which leads to hypoxia of organs and tissues, the development of multiple-organ failure and, in especially severe cases, death [269,270]. Numerous studies have shown that the development of COVID-19 is accompanied by the release of a large number of cytokines and chemokines, the spectrum of which is very broad. Thus, a group of researchers analyzed the profile of 47 signaling molecules in the plasma of COVID-19 patients in the acute phase and after recovery [271,272]. Patients diagnosed with COVID-19 had higher levels of proinflammatory cytokines (IL-6, IL-7, IL-15, IL-18, IL-27, and TNFα); chemokines (CCL2/MCP-1, CCL3/MIP-1α, CCL7/MCP-3, CCL22/MDC, CXCL8/IL-8, CXCL9/MIG, and CXCL10/IP-10); anti-inflammatory cytokines (IL-1RA and IL-10); growth factors (FGF-2/FGF-basic and G-CSF); and sCD40L. MCP-3 levels were significantly elevated in deceased patients compared to survivors [271,272]. The authors failed to identify statistically significant differences in the concentration of specific factors (IL-6, IL-15, IL-18, IL-27, TNFα, CCL2/MCP-1, CXCL8/IL-8, CXCL9/MIG, CXCL10/IP-10, IL-1RA, IL-10, G-CSF, and sCD40L) in blood plasma between the groups of survivors and deceased; however, they note that the median values in the group of deceased patients were also significantly higher [271,272]. In addition, deceased patients had higher levels of IL-1α, IFNα2, CCL4/MIP-1β, CX3CL1/fractalkine, M-CSF, and TGFα compared to controls, as noted in other reports [271,272]. The authors suggest levels of proinflammatory cytokines IL-6, IL-15, IL-18, and CXCL8/IL-8 as predictors of adverse outcome [271,272]. These data support the findings of other studies that interleukins (such as IL-1, IL-4, IL-6, IL-7, IL-10, IL-12, IL-17, and IL-18), IFN-γ, TNF-α, TGF-β, and NF-κB play an important role in the body’s inflammatory response to SARS-CoV-2 infection [273]. Other researchers have shown that levels of the cytokines IL-1β, IFN-γ, IL-10, and MCP-1 are higher in COVID-19 patients than in healthy individuals, and levels of IP-10, MCP1, MIP1a, and TNF-α are higher in ICU patients than in non-ICU patients, suggesting that overproduction of these cytokines may be associated with disease severity [274]. Finally, cytokine profiling during the acute phase of the disease showed that IL-2 and IL-12 levels were significantly higher in asymptomatic and mild patients than in severe patients, while IL-6 and IL-18 levels were significantly higher in severe patients [243]. Analyzing the presented data, it can be noted that the cytokine spectra proposed by different research groups can play a key role in the pathogenesis of COVID-19 and act as prognostic factors for an unfavorable outcome differ. This may be due to the fact that the cytokine profile can change significantly as the disease progresses. However, there are cytokines and transcription factors that most researchers note as factors that are potentially key to the development of COVID-19 pathology. These include IL-1, IL-4, IL-6, IL-7, IL-10, IL-12, IL-17 and IL-18, IFN-γ, TNF-α TGF-β, and NF-κB [268,273].

## 3. The Role of the Immune Response in the Development of Coagulopathy in COVID-19

### 3.1. Features of Coagulopathy in COVID-19

COVID-19-associated coagulopathy (CAC) is a life-threatening complication of SARS-CoV-2 infection. The development of COVID-19-associated coagulopathy contributes to morbidity and is associated with an increased risk of complications and death in patients infected with SARS-CoV-2 [5,6,8,275,276,277,278]. COVID-19-associated coagulopathy is manifested by thrombosis of large vessels and microvascular bloodstreams of the venous and arterial links of the circulatory system [279]. In addition to the lungs, it can affect other organs and systems, including the heart, brain, and kidneys [279]. Despite the enormous efforts made by researchers to establish the pathogenetic mechanisms underlying CAC, the pathogenesis of this complication of COVID-19 remains unclear. However, it is already possible to say that the pathogenesis of CAC is based on subtle interactions between components of the immune system and hemostasis which cause activation and damage to the vascular endothelium and plasma hypercoagulability [11,279]. The state of hypercoagulation and damage to the vascular endothelium and that of blood flow stasis are necessary conditions for the development of thrombosis; this id called Virchow’s triad [8]. In this section, we will consider the role of the complement system, TLR signaling, and the cytokine storm mediated by them in the development of coagulopathy in COVID-19.

### 3.2. Endothelial Activation and Damage During COVID-19

The vascular endothelium, being the largest endocrine organ in humans, plays a key role in the development of systemic disorders caused by SARS-CoV-2 infection [8]. Penetration of the virus into the body causes endothelial activation, which is expressed in increased expression of adhesion molecules, increased ROS generation, a change in anticoagulant status to procoagulant, and disruption of the barrier function of endothelial cells [280]. On the one hand, these changes can cause thrombotic complications [281]. On the other hand, they ensure the migration of immune cells outside the vascular bed, the development of an inflammatory response, and damage to organs and tissues mediated by it [281]. Histological analysis of samples of organs and tissues affected by the SARS-CoV-2 virus revealed pronounced signs of damage to vascular endothelial cells [282,283,284,285].

#### 3.2.1. Oxidative Stress

Researchers have suggested several mechanisms that might underlie vascular endothelial damage in COVID-19 [286]. One of the reasons for the impaired functional activity of endothelial cells may be excessive reactive oxygen species (ROS) production, leading to “oxidative stress”. In particular, increased ROS production is the main reason for the decrease in nitric-oxide bioavailability in cardiovascular diseases [287]. Nitric oxide is the most important vasodilator produced by endothelial cells [288]. NO also has an antithrombotic effect, inhibiting the profibrotic and prothrombotic properties of angiotensin II and endothelin I by suppressing the receptors of these molecules [289]. NO is synthesized in endothelial cells from L-arginine under the action of endothelial NO synthase (eNOS) [290]. Endothelial cells have a whole arsenal of mechanisms to protect against oxidative stress, which includes the enzymes superoxide dismutase (SOD), glutathione peroxidase, and heme oxygenase [291,292,293,294]. Excess ROS can lead to a shift in the balance towards oxidation, which ultimately leads to damage to cells and tissues.

**Figure 5:**
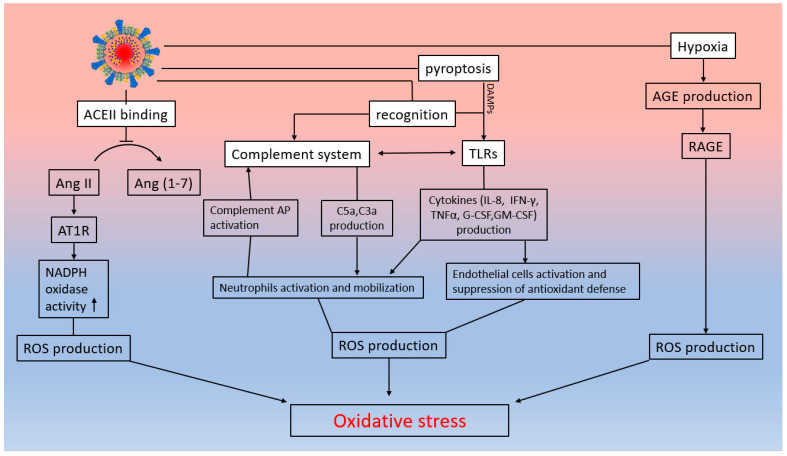
Potential mechanisms for the development of oxidative stress in COVID-19. The development of oxidative stress in COVID-19 can be mediated by several mechanisms: 1. Binding to the ACE2 receptor, the virus blocks the latter’s function of converting AngII into Ang (1–7), thereby causing the accumulation of AngII. AngII, through its receptor AT1R, stimulates the production of ROS by NADPH oxidase. 2. The virus activates the complement system and TLRs. On the one hand, this leads to the activation and mobilization of neutrophils which produce ROS. On the other hand, this can lead to activation of endothelial cells, which is accompanied by suppression of their antioxidant defense. 3. Hypoxia caused by viral pneumonia leads to an increase in the products of non-enzymatic breakdown of proteins and lipids, the end-products of which (AGE), binding to their RAGE receptor, thereby activate the production of ROS in cells.

An increase in Ang II levels and a decrease in Ang 1–7 concentrations may be one of the causes of oxidative stress. Ang II stimulates the activity of the NADPH oxidase enzyme, which is expressed in an increase in the production of superoxide anions [295]. Binding of SARS-CoV-2 to the ACE2 receptor inhibits ACE2 catalytic activity, i.e., the conversion of Ang II to Ang 1–7, thereby indirectly increasing the activity of NADPH oxidase and the level of oxidative stress in patients with SARS-CoV-2 (Figure 5) [296]. 

Neutrophils can also act as a source of ROS. Neutrophil activation and migration to the site of inflammation is due to the effect of cytokines (IL-8, IFN-γ, TNFα, G-CSF, and GM-CSF) and anaphylatoxins of the complement system (C3a and C5a) (Figure 5) [297]. In turn, TNF-α-activated neutrophils trigger the alternative complement pathway, which leads to the production of new C5a and C3a fragments [298]. Newly formed anaphylatoxins further enhance the proinflammatory responses of neutrophils, which includes ROS production [298]. This assumption is supported by the fact that increased expression of myeloperoxidase by granulocytes was detected in lung biopsy of a severely ill COVID-19 patient [299]. 

Proinflammatory cytokines can directly stimulate ROS production by the endothelial cells themselves (Figure 5). TNFα has been shown to inhibit endothelium-dependent NO-mediated dilation of coronary arterioles due to ceramide-induced activation of JNK and subsequent superoxide production by xanthine oxidase [300,301]. TNFα also inhibits NO-mediated endothelium-dependent vasodilation in small coronary arteries through activation of sphingomyelinase and subsequent superoxide radical (O_2_**^•^**^−^) production in ECs [302,303,304,305,306].

The AGE/RAGE system may contribute to the development of oxidative stress in COVID-19 (Figure 5). Hyperglycemia and oxidative stress promote the non-enzymatic oxidation of proteins and lipids [307]. The end-products of these reactions, called AGEs (advanced glycation end-products), have both direct and indirect effects on the endothelium [307]. AGE receptors (mRAGE) are expressed on the surfaces of endothelial cells, mononuclear phagocytes, and hepatocytes [308]. In addition to the form integrated into the cell membrane, there is a soluble form of RAGE (sRAGE) [309]. The interaction of AGEs with the receptor mRAGE induces ROS production, which, in addition to suppressing endogenous NO, can stimulate a cascade leading to transcriptional events induced by NF-κB. NF-κB induces TNFα expression, which may lead to further ROS production and formation of AGEs [310,311,312]. This is one of the possible pathways functioning to enhance the inflammatory response and oxidative stress. In turn, sRAGE contains only the extracellular domain and is a decoy receptor for sequestered RAGE ligands [313]. It prevents the binding of mRAGE ligands, thereby preventing the inflammatory response [313]. Type 2 diabetes mellitus (T2DM) is known to be a significant risk factor for the progression of COVID-19 and its severity, poor prognosis, and increased mortality [314]. In addition to diabetes, elevated AGE levels are characteristic of a number of other diseases that are associated with an increased risk of morbidity and mortality in COVID-19 [315]. It has been shown that in patients with COVID-19 with lung damage, the serum sRAGE level was significantly lower than in asymptomatic patients [316]. The drug metformin, designed to reduce hyperglycemia in type 2 diabetes, has shown a positive effect in the treatment of COVID-19 [317]. 

In addition to increased levels of ROS, the development of oxidative stress is also due to the suppression of the protective mechanisms of the endothelium itself. It is known that IL-6 reduces the antioxidant protection of endothelial cells, causing a decrease in the activity of the transcription factor Nrf2 and an increase in the activity of its antagonist Bach-1 [318]. Thus, the development of oxidative stress in COVID-19 can be mediated in a few ways at once: directly through the suppression of ACE2 activity and indirectly through the activation of the complement system and TLRs.

#### 3.2.2. Proinflammatory Changes in the Morphology and Adhesive Properties of the Endothelium

**Figure 6:**
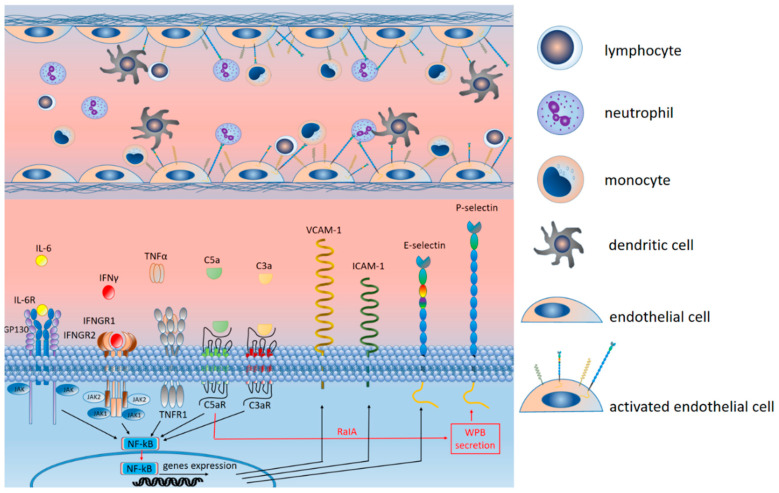
Changes in the adhesive properties of the endothelium under the influence of proinflammatory cytokines and complement anaphylatoxins (C3a and C5a). Cytokines TNFα, IL6, and IFN-γ, produced by macrophages and neutrophils, induce the expression of VCAM-1, ICAM-1, P-selectin, and E-selectin, both at the level of transcription and at the level of protein synthesis. C3a/C5a also have similar properties. Cell adhesion molecules expressed on the endothelial surface play an important role in the development of the inflammatory response, as they promote the migration of immune cells, including professional APCs (such as DCs), to the site of damage/inflammation.

The development of the inflammatory reaction is accompanied by a change in the adhesive properties of the endothelium. The inflammatory and procoagulant functions of endothelial cells are due to the activation of the expression of target genes and the exposure of the adhesion molecules encoded by them on the surface (VCAM-1, ICAM-1, E-selectin, and P-selectin) (Figure 6) [319,320,321,322]. VCAM-1 is involved in the selective adhesion of monocytes and lymphocytes [323,324]. ICAM-1 is also involved in the interaction between endothelial cells and monocytes, ensuring their adhesion and migration [325]. P- and E-selectin ensure the primary capture and rolling of leukocytes along the endothelium by forming reversible bonds with ligands on their surface [326,327]. Also, binding of selectins to P-selectin glycoprotein ligand-1 (PSGL-1) on the surface of leukocytes triggers a complex mechanism of molecular interactions leading to the activation and clustering of integrins on the leukocyte surface [326,327]. Integrins provide rolling inhibition and migration of leukocytes to the site of inflammation [326,327]. By binding immune cells and ensuring their migration to the site of inflammation, cell adhesion molecules mediate the development of an inflammatory response, which makes them potential targets for pharmacological therapy in COVID-19 [328].

#### 3.2.3. Potential Mechanisms Underlying Endothelial Adhesion and Morphological Changes in COVID-19

The mechanisms involved in changing the endothelial CAM (cell adhesion molecule) profile to a proinflammatory one are diverse (Figure 6). The expression and synthesis of adhesion molecules VCAM-1, ICAM-1, P-selectin, and E-selectin in endothelial cells can be activated by the cytokines TNFα, IL-6, and IFN-γ, which are secreted by macrophages and neutrophils (Figure 6) [329]. It has been established that the transcription factor NF-κB can play a key role in this process. The TNFα-triggered signaling pathway causes the translocation of NF-kB from the cytoplasm to the nucleus, where NF-kB activates the expression of a whole range of genes, including genes encoding CAM (Figure 6) [330]. In addition to modulating the adhesive properties of endothelial cells, the cytokines TNFα and IL-1 also disrupt the barrier function of the endothelium [331]. Morphologically, this is manifested by a conversion from a polygonal epithelial-like form of endothelial cells to a spindle-shaped organization consisting of overlapping elongated cells [332]. There is a rearrangement of actin filaments from dense peripheral strands to longitudinal stripes and a loss of fibronectin in the basement membrane [332].

In addition to cytokines, the adhesive properties of the endothelium are modeled by the complement system. Modeling can be mediated by the signaling pathways of anaphylatoxins C3a and C5a (Figure 6). In C3-/- and C3aR-/- mice, compared with wild-type mice, the levels of P-selectin, E-selectin, ICAM-1 and VCAM-1 expression in cerebral vascular endothelial cells after LPS exposure were significantly lower [333]. C3a has also been shown to activate phosphorylation of p38 mitogen-activated protein kinase (MAPK) and nuclear factor-κB (NF-κB) and induce increased expression of VCAM-1 and ICAM-1 in primary mouse-brain endothelial cells in vitro [333]. Another anaphylatoxin, C5a, has also been shown to activate expression of cell adhesion molecules (P-selectin, E-selectin, ICAM-1, and VCAM-1) [334]. In addition to anaphylatoxins, other complement factors may be involved in proinflammatory changes in endothelial cells. MASP-1, an enzyme of the lectin complement pathway, has been shown to activate endothelial cells by cleaving protease-activated receptors (PAR), resulting in cytokine production and neutrophil chemotaxis [335]. HUVEC activated by rMASP-1 decrease ICAM-2 expression and increase E-selectin expression, whereas ICAM-1, VCAM-1, and P-selectin expression remain unchanged [335]. Thus, MASP-1 can directly increase adhesion between neutrophils and endothelial cells [335]. Thus, the complement system can not only enhance TLR- and cytokine-mediated induction of proinflammatory changes in endothelial adhesive properties, but also directly trigger these changes itself.

To date, little is known about the mechanisms underlying endothelial changes in COVID-19. However, there is evidence to suggest its key role in the development of COVID-19. It has been shown that the morphology of endothelial cells of the pulmonary microvascular bed undergoes changes during COVID-19; ECs’ cellular functions are reduced, and the integrity of the intercellular barrier is disrupted [336]. Also, in COVID-19 patients, increased levels of ICAM-1, VCAM-1, VEGF, VWF, and TF in serum and plasma are observed which correlate with the severity of the disease [337]. When HUVEC endothelial cells were incubated with patient serum, increased deposition of C5b-9 on their plasma membranes was observed, compared to cells treated with the sera of healthy donors [338]. Moreover, the amounts of the C5b-9 deposits correlated with the severity of the disease, and cells treated with patient serum demonstrated increased permeability of the monolayer after treatment with the sera of deceased people [338]. Elevated C5b-9 deposits are likely due to higher complement activity in the sera of patients, which is determined by the activation of all complement pathways at once. It was mentioned above that SARS-CoV-2 can activate all three complement pathways [339,340]; their activation products were found in the lung tissue of patients with COVID-19 [205,340]. Also, increased expression levels of IL-6 and TNFα were found in the lung biopsy of a severely ill patient with COVID-19 who subsequently died [299]. Another study of biopsies from patients who died from COVID-19 showed pronounced activation of NF-κB [341]. Taken together, these data indicate that the inflammatory response in COVID-19 is accompanied by changes in the morphological and adhesive properties of the endothelium, which may be due to the complex effects of cytokines and the complement system.

#### 3.2.4. Vascular Endothelium Functions in the Regulation of Coagulation in Normal Conditions and During Inflammation

Among the functions performed by the vascular endothelium, one of the most important is the regulation of coagulation processes [342]. In addition to changes in adhesive properties and morphology aimed at increasing vascular permeability and ensuring adhesion and migration of immune cells to the site of inflammation, activation of endothelial cells leads to the change of their anticoagulant properties to procoagulant ones [342]. Under normal conditions, ECs prevent platelet aggregation and coagulation and initiate fibrinolysis, whereas in pathophysiological conditions after activation or damage they activate the blood coagulation system and promote thrombus formation [343]. 

**Figure 7:**
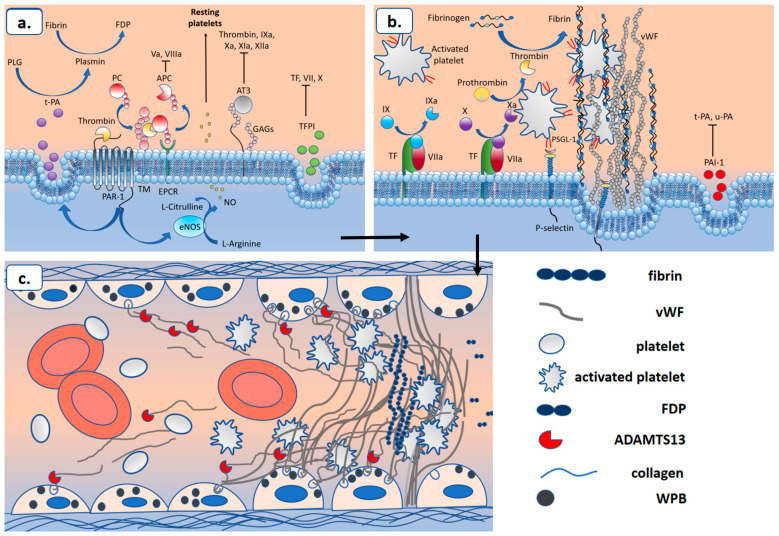
Activation of vascular endothelium and development of thrombosis. (**a**) Antithrombotic, anti-inflammatory, and profibrinolytic phenotype of endothelial cells (EC). Thrombomodulin on the EC plasma membrane binds thrombin, thereby excluding it from the blood coagulation system, and also accelerates the activation of protein C (PC), acting as a thrombin cofactor. At the same time, the endothelial protein C receptor (EPCR) binds PC and presents it to the thrombomodulin-thrombin complex for activation. Activated protein C exhibits its anticoagulant activity by binding to protein S and proteolytically inactivating FVa and FVIIIa. EC glycosaminoglycans bind antithrombin and act as its cofactor. (**b**) Activation of the endothelium during inflammation and the procoagulant state of EC. TF is expressed, as well as von Willebrand factor (vWF) and P-selectin. Multimeric vWF threads promote the formation of platelet aggregates. Increased production of plasminogen activator inhibitor-1 (PAI-1) leads to a limitation of fibrinolytic activity and an increased risk of thrombosis. P-selectin glycoprotein ligand 1 (PSGL-1), in addition to leukocytes, is expressed on platelets and promotes their interaction with the vascular endothelium during inflammation and/or hemostatic reactions. (**c**) Exocytosis of Weibel–Palade bodies leads to the release of vWF multimers in the form of threads. Normally, vWF activity is regulated by cleavage of large multimers by metalloproteinase ADAMTS-13. However, in various pathologies, there is a violation of the regulation of vWF activity, as a result of which the formation of large platelet aggregates and the development of microvascular thrombosis are observed. Such disturbances may be caused by excessive vWF release (in which ADAMTS-13 activity is insufficient) and/or ADAMTS-13 deficiency.

The anticoagulant function of the endothelium is based on several mechanisms (Figure 7). Endothelial cells carry two receptors on their surface: the thrombin receptor—thrombomodulin—and the receptor for the zymogen of the serine protease protein C (PC)—EPCR (Endothelial protein C receptor) [344]. Thrombomodulin, as a cofactor, enhances the cleavage of PC by thrombin, converting it into the active form of APC [344]. In turn, EPCR binds the zymogen PC and presents it to the thrombomodulin-thrombin complex for activation, thereby enhancing the production of the active form of APC [344].

The APC protease binds to protein S, which functions as a cofactor, and as part of the resulting complex, proteolytically inactivates blood coagulation factors Va and VIIIa [345]. The endothelial cell membrane-bound ectonucleotidase CD39 hydrolyzes ADP, a mediator that activates platelets and stimulates their aggregation, to AMP. The endothelium also secretes prostacyclin and nitric oxide, which prevent platelet aggregation and cause vasodilation. In addition to preventing thrombus formation, endothelial cells participate in fibrinolysis, the process of enzymatic degradation of thrombi, synthesizing and secreting plasminogen activators tPA and uPA [346]. 

In turn, endothelial activation during inflammation causes procoagulant changes in endothelial cells, which are expressed in increased expression of TF and overall functional activity, as well as decreased expression of adenosine triphosphate diphosphohydrolase and thrombomodulin (Figure 7) [347]. In addition, there is an increase in the secretion of von Willebrand factor (vWF) and P-selectin by endothelial cells; these are stored in Weibel–Palade bodies [348]. Von Willebrand factor is a high-molecular multimeric glycoprotein of blood plasma, which in the form of ultra-large multimers is constantly secreted by endothelial cells in quantities necessary to ensure hemostasis. One of its main functions is to ensure the attachment of platelets to the site of vascular injury with subsequent thrombus formation (Figure 7). After secretion into the vascular bed, ultra-large multimers of vWF are cleaved by metalloproteinase ADAMTS13, which leads to the formation of a heterogeneous mixture of multimers of different molecular weights. In this case, only ultra-large and large multimers of vWF have functional activity. As a consequence, increased secretion of vWF and disturbances in its metabolism are associated with the risk of thrombus formation [348].

#### 3.2.5. Potential Role of Cytokines and Complement System in Procoagulant Changes of the Endothelium in COVID-19

**Figure 8:**
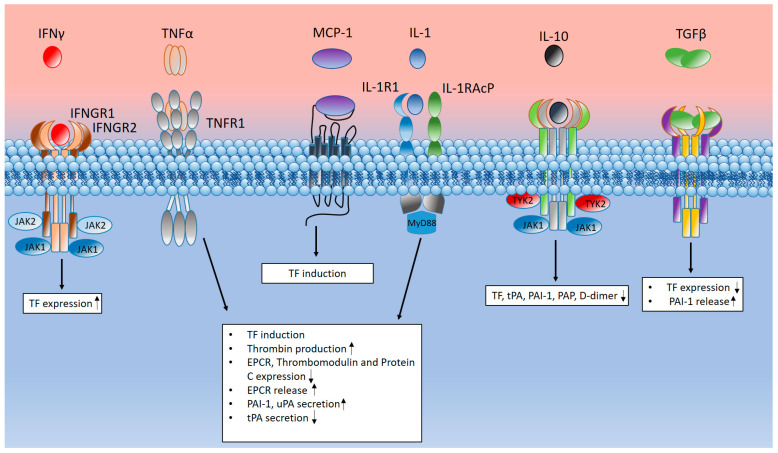
The potential role of cytokines in procoagulant changes in the endothelium in COVID-19. Cytokines TNFα, IL-1 (IL-1α and IL-1β), IFN-γ, monocyte chemoattractant protein 1 (MCP-1), etc., through interaction with their receptors, stimulate the expression of TF on the surface of endothelial cells and monocytes/macrophages, while transforming growth factor β (TGF-β) and IL-10, reduce tissue factor expression induced by various stimuli. TNF suppresses the expression of protein C receptor (EPCR), while IL-1β stimulates the release of EPCR from endothelial cells, reducing the expression of EPCR on the EC surface and, accordingly, the rate of activation of protein C. Under the influence of TNF and IL-1, thrombomodulin activity and thrombomodulin gene expression in ECs decrease. The influence of cytokines on fibrinolysis is observed: TNF, IL-1, IL-10, and TGF-β stimulate the release of PAI-1 by endotheliocytes. TNF and IL-1 (etc.) are able to increase the release of uPA, but TNF and IL-1 suppress the release of tPA by endothelial cells. The anti-inflammatory cytokine IL-10 attenuates the systemic release of tPA and PAI-1. (↓—downregulation, ↑—upregulation).

The mechanisms of endothelial transformation into a procoagulant phenotype in COVID-19 have not been fully established. On the one hand, there is reason to assume the possibility of direct induction by viral particles. In situ hybridization showed partial colocalization of SARS-CoV-2 genome RNA and TF mRNA in autopsy samples of patients who died from COVID-19. Comparison with patients whose ARDS was caused by other diseases showed that in COVID-19, the TF expression level was 2 times higher and correlated with the intensity of SARS-CoV-2 staining. According to immunofluorescence, TF protein expression was 2.1 times higher in lungs with COVID-19, compared to lungs with ARDS without COVID-19 and 11 times higher than in control samples of healthy lungs. Fibrin thrombi and platelet factor 4 (PF4)-positive thrombi were found in close proximity to TF-expressing areas in COVID-19 ARDS lungs and correlated with TF expression [349]. On the other hand, there is evidence that suggests an indirect activation of the endothelium. It has been shown that activation of vWF secretion and suppression of thrombomodulin and EPCR expression in the endothelium in COVID-19 do not require cell contact with viral particles [350]. In this case, procoagulant changes in endothelial cells may be due to their exposure to cytokines and complement factors (Figure 8). Cytokines TNF, IL-1α, IL-1β, IL-6, IL-8, leukemia inhibitory factor, IFN-γ, and monocyte chemoattractant protein 1 (MCP-1) induce procoagulant changes in endothelial cells by inducing TF expression on their surface [330,351,352,353,354,355,356,357,358,359,360,361,362,363,364,365,366,367]. In addition to activating TF expression, cytokines can suppress the activity of the protein C–protein S–thrombomodulin system. Thus, cytokines TNF and IL-1 suppress thrombomodulin gene transcription and reduce thrombomodulin activity on the surface of endothelial cells [351,353,368,369]. It has been shown that EPCR can be released from the surface of vascular endothelial cells and block the anticoagulant activity of APC [370,371]. In patients with COVID-19, the level of soluble EPCR in plasma increases already in the first days of the disease and is an early prognostic sign of probable hospitalization [372]. TNF suppresses EPCR in endothelial cells [373], while IL-1β stimulates the release of EPCR from cultured endothelial cells in vitro, reducing EPCR expression sufficiently to reduce the rate of protein C activation [371]. TNF, IL-1, lymphotoxin, IL-2, and TGF-β stimulate the release of PAI-1 by endothelial cells in vitro [357,374,375,376]. At the same time, TNF and IL-1 can also suppress the release of tPA by endothelial cells [374,375,376,377,378,379]. Also, TNF, lymphotoxin, IL-1, IL-2, and IL-4 are able to increase the release of uPA by endothelial cells [375,376,379,380,381], while IFN-γ inhibits TNF-induced uPA release [379,380]. At the same time, IFN-γ does not affect the activation of PAI-1 secretion triggered by TNF or IL-1, but can suppress the release of PAI-1 stimulated by LPS or thrombin [378]. As a consequence, the procoagulant status of the endothelium may be due to the effect of cytokines on it, the uncontrolled release of which is observed in severe forms of COVID-19.

In addition to cytokines, the complement system may be involved in endothelial activation. Receptors for anaphylatoxins C3a and C5a are also present on endothelial cells [382,383,384]. C5a promotes the retraction of endothelial cells, exposing the underlying basement membrane, and enhances tissue factor expression [385]. Sublytic MAC has a complex procoagulant effect on endothelial cells. It induces the secretion of vWF multimers and the redistribution of the platelet alpha-granule protein GMP from Weibel–Palade bodies to the plasma membrane, thereby ensuring platelet binding and thrombus formation [386]. MAC-induced secretion of vWF is accompanied by vesiculation of the cytoplasmic membrane of endothelial cells. The membrane particles formed during vesiculation carry factor-Va binding sites and maintain prothrombinase activity [387]. C4a via PAR1 and PAR4 receptors causes endothelial activation, which is associated with a decrease in its barrier functions. MASP-1 can also cause endothelial cell activation via the PAR4 receptor, which is manifested by the secretion of TF and P-selectin [388,389,390].

The complement system can activate endothelium via NETosis. NETosis is the process of formation of neutrophil extracellular traps—a chromatin complex with various cytoplasmic proteins and granule components that have antimicrobial activity [391]. NETs can be a product of one of the specialized forms of programmed cell death (classical NETosis) or be produced by cells while maintaining their viability and functional activity (vital NETosis) [391]. NETosis is a complement–dependent process [392,393]. Opsonization by complement factors C3b/iC3b enhances NETosis. This effect can be mediated by CR3 receptors. Blockade of complement receptor 3 (CR3), which binds opsonin iC3b, has been reported to inhibit NETosis in response to certain pathogens [394]. The anaphylatoxin C5a can recruit and stimulate neutrophils. The induction of NETosis by immune complexes and antibodies is significantly enhanced if they are preactivated by C5a [394]. Moreover, C5a itself can act as a NETosis stimulus [395]. In addition to C5a, NET release is promoted by C3a and MAC [18,396]. Treatment of human EC with NET increases TF expression, enhances TF activity, and accelerates the coagulation of recalcified plasma [397]. This effect was found to be due to the presence of cathepsin G in NET and is mediated by IL-1α and its receptor IL-1Ra [397]. There are several reasons for the assumption that NETosis may be involved in endothelial activation in COVID-19. Studies have shown that NETosis and NET release by circulating and infiltrating neutrophils are increased in patients with COVID-19 [398]. It has also been demonstrated that SARS-CoV-2 infection can directly induce NETosis and NET release in healthy neutrophils [398]. Elevated levels of NETosis markers are specific to patients with COVID-19 and predict the development of complications and adverse outcomes [399,400]. These data suggest that NETosis may be one of the pathways of endothelial cell activation in COVID-19.

In summary, it can be assumed that procoagulant activation of endothelial cells, along with changes in their morphology and adhesive properties in patients with COVID-19, is also determined by the complex effects of cytokines and the complement system on them, as well as complex multilevel interactions between endothelial cells and other cells functioning as mediators.

### 3.3. The Potential Role of the Complement System and the Cytokine Storm in the Development of Hypercoagulation

#### 3.3.1. Features of Hypercoagulation in COVID-19

Numerous data indicate that COVID-19 is accompanied by the development of a hypercoagulable state, which is associated with the risk of developing a wide range of thrombotic complications [277,401,402]. The incidence of such complications in severe patients can reach 31% and affect both venous and arterial blood vessels [403]. Since the identification of the first cases of COVID-19, numerous data have emerged on the increased incidence of venous thromboembolism (VTE) and arterial thromboses, such as pulmonary embolism (PE), stroke, and acute coronary syndrome [5,404,405]. The high incidence of thrombotic complications has required the inclusion of anticoagulant thromboprophylaxis in the guidelines for the diagnosis and treatment of COVID-19 [406]. The most common thrombotic events in COVID-19 are deep-vein thrombosis (DVT) and pulmonary embolism (PE) [407]. According to data presented by different research groups, the incidence of venous thromboembolism among hospitalized COVID-19 patients ranges from 12.8% to 26% [408,409,410]. Even with anticoagulant prophylaxis, the incidence of venous thromboembolism in critically ill COVID-19 patients remains high and can reach 85% [411,412,413]. A meta-analysis of 49 studies conducted in 2020 showed that the incidence of DVT among hospitalized patients diagnosed with COVID-19 was 12.1%, and PE was 7.1% [414]. A subgroup meta-analysis showed that the mean incidence of VTE was higher in studies that used ultrasound screening to detect VTE, compared to studies that used laboratory diagnostic methods to detect VTE (33.1% vs. 9.8%) [414]. These data suggest that the real incidence of VTE among hospitalized COVID-19 patients may be significantly higher than the rates reported in some studies. For example, asymptomatic DVT was detected in 85.9% of critically ill COVID-19 patients screened with ultrasound [413]. In addition to VTE, other thrombotic complications such as stroke, acute limb ischemia, and acute coronary syndrome (ACS) were observed in approximately 5–20% of hospitalized patients [415]. Moreover, the increased likelihood of developing thrombotic complications persists for some time after recovery [416]. 

Clinical studies have shown that COVID-19-associated coagulopathy (CAC) [417] is a unique syndrome that differs significantly from DIC and sepsis-associated coagulopathy [418]. In COVID-19 patients with ARDS and thrombotic complications, platelet counts are generally within the normal range and only drop below normal in severe cases [248,419]. Normal platelet counts in the blood are likely due to increased thrombopoietin production in the liver and excess platelet production by megakaryocytes in the lungs [420]. Hospitalized COVID-19 patients have been shown to have an increased immature platelet fraction [421,422]. Increases in immature platelet counts and their proportion in the total platelet fraction are associated with longer hospitalization and the progression to severe disease [421,422]. In addition to increased platelet production in COVID-19, there is the possibility of hyperactivation of platelets, which is manifested by increased surface expression of P-selectin and CD40 and increased production of thromboxane [6,423,424,425,426,427,428,429,430,431,432]. Thrombocytopenia in CAC is apparently associated with extensive thrombus formation in the lungs and correlates with mortality [419]. At the hospitalization stage, COVID-19 patients had relatively high levels of fibrinogen in the blood, which decreased in some patients shortly before death. Also, patients who subsequently died were characterized by higher PT and APTT rates [95,418,433]. 

Measurements of prothrombin cleavage product PF1+2 levels in the plasma of patients with ARDS against the background of COVID-19 showed that in severe COVID-19 patients requiring mechanical ventilation there is a powerful activation of thrombin generation, significantly exceeding that usually observed in severe sepsis and other viral infections [434,435,436]. In survivors, PF1+2 levels gradually decreased during treatment, while in patients who died, PF1+2 levels remained stable or increased slightly before death [434]. The level of PF1+2 (>500 pmol/L) can be a fairly accurate predictor of VTE development [437]. 

One important clinical indicator that is recognized as a predictor of an unfavorable outcome in COVID-19 is the fibrin degradation product—D-dimer. An elevated level of D-dimer during hospitalization of COVID-19 patients is a poor prognostic marker and is associated with a severe course of the disease and in-hospital mortality. Multivariable regression analysis of 191 patients with COVID-19 showed an odds ratio of 18.42 (2.64–128.55, *p* = 0.0033) for in-hospital mortality for patients with D-dimer > 1 μg/mL [276]. D-dimer levels > 2000 ng/mL were associated with the highest risk of thrombotic complications, acute kidney injury, critical illness, and death [438]. This suggests that fibrinolytic activity increases in COVID-19 patients against the background of hypercoagulation. However, clinical analyses of the fibrinolytic system, fibrinolysis inhibitors, and endogenous anticoagulants indicate otherwise. Numerous studies have shown a decrease or even a virtual absence of fibrinolytic activity in patients with severe COVID-19 [439,440]. There is evidence that COVID-19 patients develop resistance to exogenous fibrinolytics [441,442,443], which may be due not only to the suppression of factors involved in plasminogen activation, but also to increased activity of fibrinolysis inhibitors. Indeed, it has been established that the development of a severe form of COVID-19 is accompanied by inhibition of tissue plasminogen activator (tPA) secretion, up to its complete suppression [434]. At the same time, the levels of the fibrinolysis inhibitor PAI-2 and the plasmin–antiplasmin complex PAP in patients with COVID-19 with ARDS, on the contrary, increase [434]. The authors of the study showed that the PAI-2/PAP ratio can be one of the predictors of death in COVID-19 [434]. Data are presented that in patients with COVID-19 admitted to the ICU, the levels of plasminogen activator inhibitor-1 (PAI-1) and thrombin-activated fibrinolysis inhibitor (TAFI) are increased and a high ability to generate thrombin is observed, which was maintained even while taking heparin [444]. It has been reported that PAI-1 levels > 10.2 ng/mL (sensitivity 83%, specificity 83%) and >TAFI 818.05 ng/mL (sensitivity 83.3%, specificity 71.4%) in the plasma of COVID-19 patients predict mortality [445,446]. Tang et al. reported that plasmin-alpha-2-antiplasmin (PAP) complexes and tPA-PAI-1 were elevated in COVID-19 non-survivors, indicating a state of profound fibrinolytic suppression [95]. 

The dynamics of changes in endogenous anticoagulant levels were studied in patients with ARDS due to COVID-19 with organ failure scores of SOFA ≤ 10 and SOFA > 10. At the time of admission to the ICU, all patients had plasma plasminogen, protein C, and antithrombin levels within the normal range, and a decreased level of free protein S. Over time, the plasma antithrombin level in patients with SOFA ≤ 10 slightly increased, while it remained at the same level in patients with SOFA > 10. Protein C levels increased throughout the observation period in both groups, but were consistently higher in the SOFA ≤ 10 group. Plasma free protein S levels increased over time without differences between the groups and reached the normal range [447]. Another group of researchers, in addition to a decrease in protein S in patients hospitalized with a diagnosis of COVID-19, observed a decrease in protein C, a decrease in factor XII, and a decrease in antithrombin activity.

Taken together, these data indicate that activation of the coagulation system in COVID-19 is accompanied by suppression of the fibrinolytic system and the endogenous anticoagulant system and increased activity of fibrinolysis inhibitors in the circulatory system. At the same time, the question of the reasons for the increased D-dimer level in the blood of COVID-19 patients remains open. The most likely assumption is that the increase in D-dimer levels in COVID-19 patients is due to local, extravascular activity of the fibrinolytic system in the lungs and may not reflect the degree of activation of plasminogen and fibrinolysis occurring in tissues [448,449]. This assumption was partially confirmed by a comparative analysis of the activity of the blood coagulation system and the complement system, the composition of cytokines and chemokines in bronchoalveolar lavage fluid (BALF) and blood plasma samples of patients with severe COVID-19 [450,451]. The study data showed that both coagulation activation and fibrinolysis activation occur in the lungs of COVID-19 patients. In the BALF of COVID-19 patients, significantly higher levels of soluble tissue factor, thrombin–antithrombin complex (TAT), plasminogen activator tPA, PAI-1, and D-dimer were detected, compared to the control group of healthy people. The most pronounced differences were observed in the levels of D-dimer and TAT. A different picture was observed in the blood plasma of COVID-19 patients. While the level of D-dimer in the plasma of patients was also significantly elevated, the levels of soluble tissue factor and TAT remained within the normal range, and the differences in the level of tPA and PAI-1 in the plasma of patients and the control group were significantly less pronounced [450]. These data suggest that in patients with severe COVID-19, local activation of coagulation is more pronounced compared to systemic activation. At the same time, the absence of significant differences in the tPA/PAI-1 ratio in BALF between COVID-19 patients and healthy donors indicates that elevated D-dimer levels likely reflect increased coagulation rather than the development of hyperfibrinolysis. 

Coagulopathy in COVID-19 is characterized also by elevated levels of factor VIII and von Willebrand factor (vWF) and decreased ADAMTS13 activity [452,453]. Impaired vWF metabolism underlies various thrombotic microangiopathies, such as STEC-HUS, aHUS, and TTP, leading to the development of organ failure and death. TMA is one of the forms of thrombotic disorders observed in patients with COVID-19 [454,455]. We have shown that decreased ADAMTS13 activity is a predictor of poor outcome in COVID-19 patients with kidney damage.

#### 3.3.2. The Potential Role of Cytokines in the Development of Hypercoagulation in COVID-19

We have already described above how cytokines can change the properties of endothelial cells, causing their morphological changes, exposure of adhesion molecules and tissue factor, and secretion of vWF. The data presented in the literature indicate that the function of cytokines in regulating hemostasis is not limited to the effect on the endothelium. Numerous studies have shown that cytokines are involved in the regulation of a whole range of hemostasis elements. In this case, cytokines can both stimulate and suppress the activity of the blood coagulation system. 

Cytokines that have a stimulating effect on the blood coagulation system include TNF, IL-1α, IL-1β, IL-6, IL-8, leukemia inhibitory factor (LIF), IFN-γ, and monocyte chemoattractant protein 1 (MCP-1). In particular, it has been shown that these proinflammatory cytokines exhibit the ability to activate TF expression in endothelial cells and/or monocytes/macrophages [351,352,353,354,355,356,357,358,359,360,361,362,363,364,365,366,367,456]. In turn, transforming growth factor (TGF)-β, IL-4, IL-10, and IL-13, which are classified as anti-inflammatory cytokines, on the contrary, suppress the expression of TF mediated by the effect of various pathogens and endogenous proinflammatory factors (C-reactive protein, LPS, TNF, IL-1, and MCP-1) on cells [355,356,357,358,361,362,363,457,458]. In vivo studies have demonstrated that TNF, IL-1, IL-6, IL-12 and IL-2 can induce thrombin production in humans and primates. In turn, IL-10 can act as an anticoagulant cytokine, but this effect is most likely mediated by suppression of the release of proinflammatory cytokines [459,460]. 

In addition to activating TF expression, cytokines regulate the activity of the protein C-protein S-thrombomodulin system. EPCR can be released from the vascular endothelium, and soluble EPCR can block the anticoagulant activity of APC [370]. TNF and IL-1 reduce thrombomodulin activity and gene expression in endothelial cell cultures [351,353,368,369]. TNF suppresses EPCR in endothelial cells [373], while IL-1β stimulates EPCR release from cultured endothelial cells in vitro, reducing EPCR expression sufficiently to decrease the rate of protein C activation [371]. Studies in mice indicate that TNF and IL-1 may enhance the procoagulant response to sepsis by suppressing protein C synthesis at the tissue level, i.e., injection of recombinant TNF into mice resulted in decreased protein C mRNA expression in the liver, kidney, and testes; IL-1 administration was associated with decreased protein C mRNA expression in the liver and testes, but not the kidney [461]. 

TNF, IL-1, lymphotoxin, IL-2, and TGF-β stimulate PAI-1 release by endothelial cells in vitro [357,374,375,376]. At the same time, TNF, IL-1 can suppress tPA release by endothelial cells [374,375,376,377,378,379]. Also, TNF, lymphotoxin, IL-1, IL-2, and IL-4 are able to increase uPA release by endothelial cells [375,376,379,380,381], while IFN-γ inhibits TNF-induced uPA release [379,380]. However, IFN-γ does not affect the activation of PAI-1 secretion triggered by TNF or IL-1, but can suppress PAI-1 release stimulated by LPS or thrombin [378]. In addition to the endothelium, other cell types can be cellular targets, including hepatocytes, monocytes, fibroblasts, and keratinocytes [462,463,464,465]. Infusion of recombinant TNF into human volunteers caused an initial activation and subsequent inhibition of plasmin generation. TNF has also been shown to mediate the fibrinolytic response to LPS administration in humans and chimpanzees [460,466,467]. Infusion of IL-1α into baboons was also associated with an early activation of the fibrinolytic system, with a characteristic subsequent increase in plasma tPA and PAI-1 concentrations that was suppressed by recombinant IL-1ra [468]. IL-2 has been shown to stimulate the release of tPA and PAI-1 in cancer patients [469]. Injection of IL-12 into chimpanzees causes a delayed fibrinolytic response characterized by an increase in plasma tPA and PAP complexes and no change in PAI-1 levels [470]. In turn, IL-10 attenuates the systemic release of tPA, PAI-1, PAP, and D-dimer complexes activated by LPS exposure [459]. The involvement of IL-6 in fibrinolytic activity has not yet been confirmed [466,471]. 

It is important to note that the interactions between cytokines and the blood coagulation system are bidirectional, since hemostatic mediators are also able to influence the activity of cytokines. The end-products of the coagulation system can cause inflammatory reactions in vitro. Thrombin, prothrombin, and factor Xa have been shown to enhance LPS-induced activity of IL-1 produced by guinea pig macrophages [472]. Factor Xa, thrombin, and fibrin can also activate endothelial cells, causing the synthesis of IL-6 and/or IL-8. [473,474,475,476]. Thrombin also increases IL-8, MCP-1, E-selectin, and PAI-1 mRNA levels in cultured endothelial cells and enhances TNF-induced E-selectin expression [477]. In addition to endothelial cells and macrophages, thrombin can stimulate the cytokine response of monocytes [478,479]. In turn, activated protein C can exert an anti-inflammatory effect on LPS-stimulated monocytes/macrophages by suppressing the release of TNF, IL-1b, IL-6, and IL-8. This effect can be synergistically enhanced by the addition of protein S [480,481,482,483]. Furthermore, APC is not only able to reduce cytokine production by monocytes in vitro, but also to reduce LPS-induced TNF release in rats in vivo [480,482,483]; additionally, anti-APC antibodies enhance TNF secretion when rats are exposed to LPS [483]. In turn, anti-EPCR antibodies enhance the release of IL-6 and IL-8 in baboons with sublethal *Escherichia coli* bacteremia [484]. PAI-1 inhibits LPS-induced TNF production by mononuclear cells in vitro [485,486]. In contrast, uPA enhances LPS-induced TNF secretion through an effect independent of plasmin activity [487]. In fact, uPA may also regulate cytokine activity, as evidenced by the finding that U937 monocytic cells can produce cell-associated uPA, which is capable of inactivating IFN-γ by proteolysis [487]. 

These data indicate that cytokines may mediate SARS-CoV-2 infection-induced changes in hemostasis and the development of thrombotic complications, not only by activating endothelial cells and stimulating activation of the coagulation system, but also by affecting the fibrinolytic system and the endogenous anticoagulant system. The nature of these changes is likely due to the balance in cytokine activity, interactions between them, and effects on different cell types. The TF-FVIIa pathway, which likely plays a dominant role in coagulation activation in COVID-19, may have a proinflammatory function that is not directly related to the coagulation cascade, as has been shown in other infection-associated hemostatic disorders [484,488,489,490,491,492]. In turn, the protein C-protein S-thrombomodulin system not only suppresses coagulation, but may also have an anti-inflammatory effect. The fibrillolytic system may also be involved in the regulation of inflammation in COVID-19. Cytokine-mediated imbalance in the activity of these elements that ensure hemostasis may be one of the mechanisms for the development of pathologies in COVID-19 and determine the severity of the disease. The following facts indirectly support these assumptions. As described above, patients with severe COVID-19 are characterized by increased activity of the coagulation system and suppression of the fibrinolytic system and the protein C–protein S—thrombomodulin system in blood plasma. At the same time, they have an increased level of proinflammatory cytokines. A comparative analysis of cytokines in BALF and blood plasma of severe patients with ARDS against the background of COVID-19 showed increases in the levels of TNF-α, IL-1α, IL-1β, IL-1ra, IL-6, IL-10, and IL-33 cytokines relative to healthy donors [450]. These studies have shown that the local increase in cytokine levels in BALF was significantly higher than in blood plasma [450]. Considering that the local activity of the blood coagulation system in the lungs in these patients was higher than the systemic activity, it seems logical to assume that cytokines are involved in pathogenetic changes in the activity of the blood coagulation system in COVID-19 [450]. Dynamic observations have shown that these patients experience a significant decrease in TNFα, IL-1α and IL-1β in BALF, which is accompanied by decreases in sTF, tPA, sCD40L and sP-selectin [450]. In blood plasma, the changes were of a different nature. There, decreased levels of IL-RA, IL-1b, and IL-6 IL-10 were detected, which was accompanied by decreases in TAT and PAI-1. Moreover, in patients with pulmonary embolism, compared to patients without it, on the one hand, IL-6 in plasma was increased, while on the other hand, IL-10 in BALF was decreased [450].

#### 3.3.3. Interactions Between the Complement and Coagulation Systems and Their Potential Rolest in Hypercoagulation in COVID-19

The complement system and the blood coagulation system, the main proteolytic cascades of the circulatory system, jointly provide protection of the body during injuries and pathologies through numerous biochemical interactions [493]. Their coordinated work leads to the stopping of bleeding, the reduction of the spread of the pathogen and its removal from the body, and the facilitation of wound healing [493]. At present, complement and coagulation are considered as inextricably linked phenomena. In addition to their origins, these two systems have common cellular targets, and factors of one system can be activators or inhibitors of the other [390]. There are many molecular interactions between complement and the blood coagulation system which are coordinated through feedback mechanisms [390]. The development of thrombohemorrhagic syndrome against the background of septic complications of viral and bacterial infections illustrates the close interaction between complement and coagulation [494]. It is suggested that hyperactivation and/or dysregulation of the activity of one system disrupts the delicate mechanism of operation and interaction pathways between the two cascade systems, which may ultimately lead to the development of the severe thrombotic complications observed in COVID-19 [495].

Since activation of the complement and coagulation systems is observed in COVID-19, it seems logical to assume that the hypercoagulable state in COVID-19 may be at least partially due to positive feedback between these two systems. Moreover, these interactions can simultaneously stimulate coagulation and increase inflammation. Several facts support this assumption. As we noted above, activation of the complement and coagulation systems is observed in COVID-19 patients, which is accompanied by suppression of the fibrinolytic system and endogenous inhibitors of the blood coagulation system. Pronounced activation of the complement system was observed only in cancer patients with COVID-19 who had elevated D-dimer [496]. Moreover, a direct correlation was found between D-dimer and complement-activation markers C3a, C5a, and sC5b-9, and an inverse correlation between the D-dimer level and the level of complement inhibitor C1 INH [496]. Also, an increase in platelet and endothelial activation markers was noted in cancer patients with COVID-19 [496]. Genetic studies have revealed a correlation between mutations in the genes encoding complement and blood coagulation system regulators and the severity of COVID-19 [497]. Analysis of endogenous thrombin potential (ETP) in the plasma of COVID-19 patients showed that severe complications are associated with the development of resistance to the blood coagulation system inhibitor thrombomodulin (TM) [498]. It was shown that an increased level of sTM in blood plasma correlated with the severity of the disease [499]. Autopsy analysis of lungs from patients who died from COVID-19 revealed downregulation of TM and EPCR expression in lung endothelial cells, which was associated with severe coagulation impairment, immune cell infiltration, and platelet activation [350]. Low plasminogen levels were also shown to be significantly associated with inflammatory markers (CRP, PCT, and IL-6), coagulation markers (D-dimer, INR, and APTT), and organ dysfunction markers (high fasting blood glucose and decreased glomerular activity and filtration rate) [500]. The hypercoagulable state in COVID-19 patients is at least partially mediated by activation of the contact pathway of the coagulation system [501]. In patients with severe COVID-19, the activity of the contact pathway of the coagulation system can be enhanced by the complement system through the recruitment of neutrophils and NETosis [501]. 

At least in part, the activation of complement and the blood coagulation system and disturbances in their regulation may be mediated by the direct effects of viral particles on these systems. It has been shown that viral structural proteins can bind various proteins involved in the activation of the complement system, the kallikrein-kinin system, and the blood coagulation system [502]. Thus, activation of the complement system can be mediated by direct binding of viral proteins to C1q and its receptor gC1qR [502]. In turn, binding of SARS-CoV-2 structural proteins to gC1qR, FXII, and high-molecular-weight kininogen can activate the kallikrein-kinin system [502]. It has been shown that gC1qR can interact with various factors that ensure hemostasis, including fibrinogen, thrombin, FXII, and multimeric vitronectin [202]. Based on these data, it can be assumed that by binding gC1qR, the virus can also moderate the activity of the blood coagulation system. The data provided indicate the presence of potential interactions between complement and the blood coagulation system which may cause the development of hypercoagulability syndrome in patients with COVID-19.

#### 3.3.4. Mechanisms of Interaction Between the Complement and Coagulation Systems, That May Be Involved in Hypercoagulation in COVID-19

We have discussed above the activation of the endothelium by complement factors and the acquisition of procoagulant status by endothelial cells. In addition to the vascular endothelium, other cells, immune and non-immune, are exposed to the complement system and are also involved in enhancing inflammation and activating coagulation. Platelets play a key role in these processes. Endothelial damage causes platelet binding by vWF and their activation by agonists such as subendothelial matrix proteins and ADP [503,504,505,506]. Platelet activation causes exposure of complement anaphylatoxin receptors C3a and C5a on their surface [507]. In turn, activation of complement anaphylatoxins C5a and C3a receptors C5aR and C3aR on the platelet surface causes their aggregation and secretion of intracellular granules [508,509,510]. Platelets contain granules of different types. The largest are α-granules. They contain more than 300 different proteins and peptides, among which are adhesion molecules (for example, P-selectin (CD62P)), growth factors, cytokines and chemokines (for example, PF4, which neutralizes heparin-like molecules on the endothelial surface of blood vessels), blood coagulation factors and inhibitors (for example, TFPI), fibrinolysis inhibitors (PAI-1), complement components and associated regulators, vWF, platelet activating factor (PAF), and chondroitin sulfate [511]. In turn, dense granules contain the monoamines serotonin and histamine and nucleotides ADP, ATP, and GTP, as well as Ca^2+^ and Mg^2+^ [390,512]. Molecules secreted by platelets have a wide range of activities [511]. In particular, they enhance platelet activation, activate the vascular endothelium, mobilize immune cells, and stimulate complement activity, thereby creating conditions for the development of an inflammatory response and coagulation [513,514,515]. It has been shown that sC5b-9 is also capable of activating platelets [516]. Platelets are known to contain complement components (C1q, C3(H_2_O), C4, properdin, factor D, C8, C9, and, to a lesser extent, C5, C6, and C7) [516]. 

After their own activation, platelets are intensively involved in the activation of the complement system and the blood coagulation system, using several mechanisms at once. The complement proteins they secrete are included in activation cascades. Molecules that enhance the complement cascade are integrated into the platelet membrane, binding its components and mediating the activation of the classical and alternative pathways [512]. Thus, P-selectin, by binding C3b to the platelet surface, stimulates activation of the alternative pathway, whereas the gC1q-R receptor and chondroitin sulfate on the surface of activated platelets can trigger the classical signaling pathway, acting as ligands for C1q [517,518,519]. In turn, C1q not only mediates activation of the classical pathway, but can also cause relatively rapid exposure of P-selectin on the platelet surface, modulating the activation by collagen [520]. Activation of the alternative pathway on the surface of activated platelets can be mediated by properdin. By binding to the platelet surface, it recruits C3(H_2_O) and C3b molecules, ensuring the assembly of C3 convertases of the alternative pathway [521,522]. By actively participating in complement activation, platelets have an arsenal of tools that protect them from complement-mediated lysis. They secrete, expose on their surface, and accumulate from blood plasma a whole complex of regulatory factors of the complement system, which includes C1-INH, FH, C4BP, MCP, DAF, and Protectin [516]. The addition of sC5b-9 to platelets and the assembly of MAC on their surfaces not only does not cause lysis, but leads to the activation of platelets and the secretion of α-granules containing complement proteins and factor V [523]. Also, the integration of MAC into the platelet membrane stimulates the assembly of prothrombinase and the release of membrane microvesicles with prothrombinase activity [523,524,525]. It was shown that the density of MAC and sites of formation of prothrombinase complexes FXa/FVa on the surface of microvesicles is significantly higher than on the platelet membrane [525]. These data suggest that the process of microvesicle release on the one hand protects platelets from the lytic activity of MAC, and on the other hand can enhance the activity of the coagulation system in the circulation. Furthermore, microvesicles support the activity of the complement system through activation of the classical pathway [526]. 

In addition to platelets, the complement system closely interacts with immune cells, the activation of which can also lead not only to the development of an inflammatory reaction, but also to hemostasis disorders. Thus, C3a and C5a, similar in their three-dimensional structure, are known as effective chemotactic molecules and mediators of immune cell activation [169,396,527]. C3a and C5a exert their physiological and pathophysiological effects mainly through C3aR and C5aR1, but C5a can also bind to C5aR2 [528]. C3aR and C5aR1 are expressed by many immune cells. These include mast cells, neutrophils, and monocytes/macrophages [528]. According to their biological activity, anaphylatoxins can be arranged in the following order: C5a > C3a > C4a [529]. 

We have previously reported that C3a, C5a, and MAC promote the recruitment and activation of neutrophils [18,394,395,396]. In addition to stimulating NETosis, the interaction of C5a with its receptor C5aR1 (CD88) results in the expression of tissue factor (TF), the initiator component of the extrinsic pathway of the blood coagulation cascade, on the surface of neutrophils [530]. NETs released by neutrophils also contain functionally active TF [18,396]. NETs bind plasma proteins such as fibronectin and vWF and, as a scaffold, promote the aggregation and adhesion of platelets and erythrocytes and further stabilize the clot [531,532]. It has been reported that the procoagulant effects of NETs are independent of FXI, FXII, or FVII, and are likely mediated by FXI and, to a lesser extent, FXII [531]. In this case, NETs can apparently activate FXII, the initiator of the intrinsic coagulation pathway, via negatively charged extracellular DNA [533]. In addition to their involvement in coagulation, NETs are a site for activation of the complement system, creating positive feedback and enhancing the effects of complement [534]. Active enzymes and factors released by neutrophils can also influence thrombosis. Cathepsin G and elastase are known to activate platelets and factors V, VIII, and X and cleave tissue factor pathway inhibitor (TFPI), which leads to increased procoagulant activity [535,536,537,538,539,540,541]. Elastase also inactivates antithrombin III and, depending on the concentration, can lead to an almost complete loss of the latter’s functional activity in relation to thrombin [542]. Studies on the role of neutrophils in the development of thromboinflammation in COVID-19 have confirmed that complement activation enhances the platelet/NETs/TF/thrombin axis during SARS-CoV-2 infection [18]. Moreover, blocking C3a has shown greater efficacy in preventing NET-induced thromboinflammation in COVID-19 compared to C5a [543].

In human mast cells and basophils, C5a induces the production of plasminogen activator inhibitor-1 [544]. Resting mast cells constitutively express tissue plasminogen activator (t-PA), which retains its enzymatic activity in the absence of PAI and promotes plasmin formation [545]. Thus, in contrast to the norm, under the influence of C5a, mast cells acquire an antifibrinolytic, prothrombotic phenotype. These interactions also highlight the close relationship between coagulation and innate immunity. Activation of macrophages by anaphylatoxins C3a and C5a activates the NLRP3 inflammasome, causing the release of proinflammatory cytokines and ROS production [546]. In turn, activation of the NLRP3 inflammasome can promote coagulation through activation of TF expression [547]. 

Thus, platelets and immune system cells can significantly contribute to the development of inflammation and hypercoagulation in COVID-19 patients, acting as an intermediary between the complement system and the blood coagulation system. Furthermore, factors of one system can directly influence factors of another system. Let us consider several examples. Complement fragments can promote thrombin formation or perform its function. Thus, serine proteases MASP-1 and MASP-2 can activate prothrombin to form thrombin [548,549]. MASP-1, like thrombin, cleaves fibrinogen to fibrin monomers and activates factor XIII and the thrombin-activated fibrinolysis inhibitor TAFI [493,550]. C3 is reported to promote fibrin clot stability and its resistance to fibrinolysis is due to its ability to bind to both fibrinogen and fibrin [551,552]. This binding can be mediated by FXIIIa [553]. Complement inhibitors have a significant effect on the blood coagulation system. For example, many interactions have been found between factor H and factors of the blood coagulation system, which is the subject of a separate review article [554]. Factor H binds to blood coagulation factors FXI, FXII, FXIII, and thrombin, as well as to fibrinogen and fibrin within the fibrin clot, which has functional significance for both the complement system and the blood coagulation system [554]. C1-esterase inhibitor (C1-INH), which blocks proteases C1r, C1s, MASP-1, and MASP-2, and also directly binds C3b, thereby suppressing the formation of C3-convertases CP/LP/AP, is the main regulator of the plasma kallikrein-kinin system, since it is able to inactivate activated plasma kallikrein, factor XIIa, and factor XIa [548,555]. C1-INH also inhibits plasmin and tissue plasminogen activator (tPA) [184,556,557,558,559].

The interaction of the complement system and the blood coagulation system also works in the opposite direction: factors of the coagulation and fibrinolysis cascade, as well as the kinin-kallikrein system, in turn, are capable of exerting an activating or inhibitory effect on the complement system. Some factors, such as thrombin and thrombomodulin, can play a dual role, since they can act both as complement activators and as inhibitors. For example, kallikrein can participate in AP, since it is able to cleave factor B, forming Bb and Ba [560]. It also activates the complement system by cleaving C3 and generating active fragments C3b and C3a. In this case, kallikrein recognizes the same cleavage site within C3 as C3 convertase [561]. Activated Hageman factor (XIIa) can initiate the classical pathway of complement activation through interaction with the C1 complex: activation of C1r and further activation of C1s by C1r, and, to a lesser extent, by its direct action [557,562]. A number of proteases of the blood coagulation system exhibit activity towards C3 and C5, promoting their cleavage and formation of functional anaphylatoxins C3a and C5a, respectively. Thrombin, plasmin, FXIa, FIXa, and, the most effective in this regard, FXa have this function [557,563]. It is assumed that in the genetic absence of C3, thrombin can replace the C3-dependent C5 convertase [181]. Thrombin action on protease-activated receptors (PARs) on the platelet plasma membrane induces C3 and MAC deposition [564,565]. Studies investigating the role of MASP-3 in pro-FD activation suggest that thrombin or thrombin-activated proteases may act as a backup activator of pro-FD [566,567]. Thrombin has also been reported to cleave C5 at site R947, generating previously undescribed intermediates C5(T) and C5b(T) and promoting the formation of a MAC-like complex (C5b-9T) with significantly increased lytic activity [568]. Thrombomodulin affects C3 activation of the alternative complement pathway, in a manner presumably similar to that of properdin [569]. 

The following factors can act as complement inhibitors: plasminogen/plasmin, thrombin, antithrombin, TAFI, tissue factor pathway inhibitor (TFPI), and thrombomodulin. It is known that plasminogen is able to bind to C3, C3b, C3d, and C5 via lysine residues [570]. In doing so, it enhances the cofactor activity of factor H and promotes the cleavage of C3b by factor I. The active form of plasminogen, plasmin, cleaves C3b and C5, and the resulting fragments differ from those generated by factor I [570]. Thrombin plays an important role in regulating complement and protecting the endothelium from damage caused by increased C3 binding. It binds to PAR1 on the surface of ECs and triggers the synthesis of DAF (CD55) de novo [571]. Antithrombin, which controls the activity of thrombin, FXa, and FIXa [572,573], is an effective inhibitor of the lectin pathway, as it can suppress MASP-1 and MASP-2, and is especially effective in the presence of heparin [574]. Tissue factor pathway inhibitor (TFPI), which binds factor Xa (FXa) and in complex with it inhibits the activity of TF complex with FVIIa (TF-FVIIa) [575,576,577], is also able to inhibit the lectin pathway by interfering with the activity of the MASP-2 protease [578]. Thrombin-activated fibrinolysis inhibitor TAFI, after activation by the endothelial thrombin/thrombomodulin complex, inactivates C3a and C5a, exhibiting greater catalytic activity compared to plasma carboxypeptidase N (CPN) [579]. Inactivation occurs due to specific cleavage of the carboxyl terminal arginines [579]. The role of proCPB has also been confirmed in vivo experiments, since mice deficient in proCPB have been shown to exhibit increased pulmonary inflammation in a model of C5a-induced alveolitis [580]. Thrombomodulin, in addition to activating complement, can also suppress it. It enhances the degradation of C3b to iC3b by factor I in the presence of its cofactor, factor H [569,581,582]. Thus, the blood coagulation system and the complement system have common activators and inhibitors that ensure their coordinated work. Factor XI in its active form has an inhibitory effect on one of the main regulatory proteins of the complement system, factor H [583]. FXIa reduces the functional activity of FH, thereby suppressing the cleavage of the alternative pathway C3 convertase and inactivation of C3b and prevents the binding of FH to the vascular endothelium [584]. In turn, FH affects the components of the blood coagulation system [554]. The same article mentions the inhibitory effect of factor H on the activation of FXI by thrombin or FXIIa. It is known that factor H is able to bind thrombin and act as its cofactor, enhancing the pro- and anticoagulant effects of thrombin [554]. Factor H is able to form a complex with FXIIa [585]. In addition, the existence of a functional interaction between factor H, VWF, and ADAMTS-13 has been noted. FH binds to VWF and enhances its proteolysis by the ADAMTS13 enzyme, presumably by changing the conformation of the molecule and facilitating the accessibility of cleavage sites [586,587,588,589]. In turn, ADAMTS13 cleaves large multimers of vWF, thereby activating its cofactor activity in the cleavage of C3b molecules by factor I [590]. 

The relationship between the blood proteolytic systems is supported by in vivo experiments. It has been previously reported that proCPB-deficient mice exhibit increased pulmonary inflammation in a C5a-induced alveolitis model [580]. However, mutant thrombin E229K, which has reduced procoagulant properties but retains the ability to activate protein C and TAFI, reduces the severity of alveolitis in wild-type mice but not in TAFI-deficient mice [580]. Impaired platelet aggregation, prolonged bleeding time, recurrent tail-cut bleeding, and thrombus instability have been reported in C3-/- mice compared to wild-type mice [61]. Later experiments in C3-/- and C5-/- mice showed that deficiency of anaphylatoxins C3 and C5 have different effects on thrombus formation: C3 deficiency reduces platelet activation, while C5 deficiency impairs TF- and myeloid cell-dependent fibrin formation in venous thrombosis and does not affect platelet activation [61,591]. The function of the blood coagulation system is also affected by the absence of factors of the lectin pathway of complement. Thus, in mice with knockout of MBL or MASP1/3, an increased bleeding time is observed during tail tip excision, as well as a decrease in FeCl3-induced thrombus formation [592]. Infection of MBL-null mice with gram-positive Staphylococcus aureus leads to the development of DIC syndrome with high mortality compared to wild-type mice [593]. Thus, a large amount of data has accumulated emphasizing the interactions and interdependence between the complement system and other blood cascade systems. 

Summarizing all of the above, it can be concluded that the complement system is capable of activating the blood coagulation system in a few ways: (1) activation of ECs, platelets and other immune and non-immune cells, as a result of which the tissue factor pathway of the blood coagulation cascade is launched and the transition of cells to a prothrombotic, antifibrinolytic phenotype is observed; (2) direct activation of the coagulation system factors. In turn, activation of the coagulation cascade enhances inflammation and the activity of the complement system, while inhibitors of the coagulation system and the fibrinolytic system, on the contrary, perform an anti-inflammatory function suppressing the effects of complement. Apparently, there is a complex network of direct and mediated interactions between the complement system and the blood coagulation system. Disturbances in the activity of one system, as well as changes in the complex mechanism of complement–coagulation system interactions, can lead to serious changes in the other. Such disturbances can be one of the main reasons for the development of severe complications in COVID-19.

## 4. Therapeutic Approaches for COVID-19

COVID-19 has become a serious challenge for humanity, affecting all areas of life and placing a heavy burden on the healthcare system. Enormous efforts are being made to study the mechanisms of COVID-19 pathogenesis and find effective drugs for its treatment. At the very beginning of the pandemic, therapy with the direct-acting anticoagulant low-molecular-weight heparin was introduced into COVID-19 treatment protocols, and demonstrated limited effectiveness in the treatment of severe patients [594,595,596]. Thus, low-molecular-weight heparin therapy significantly reduced the risk of developing deep-vein thrombosis in COVID-19 patients, but did not exclude it completely. [594]. In addition to heparin, vitamin K-independent factor Xa inhibitors (NOACs), such as rivaroxaban and analogues, have been used [597]. These drugs have shown their high efficacy and safety in thromboprophylaxis in various diseases [598,599]. However, the higher efficacy of NOACs compared to heparins remains a matter for further research [597]. 

Studies have shown that various systems of innate and adaptive immunity are involved in the pathogenesis of COVID-19 [600,601]. These data formed the basis for the assumption that immunothrombosis develops in COVID-19 and significantly expanded the range of potential targets for pharmacological therapy [494]. Glucocorticosteroids, which have potent non-specific anti-inflammatory and immune system suppressive effects, have found their use in the treatment of COVID-19 [602]. In addition to broad-spectrum drugs, targeted therapy has begun to be used more actively [603]. Thus, the treatment program began to include complement inhibitors (e.g., eculizumab, ravulizumab, and zilucoplan), SGLT-2 inhibitors with anti-inflammatory activity (empagliflozin), Janus kinase inhibitor JAK1 and JAK2 baricitinib, humanized monoclonal antibodies against cytokines and their receptors (tocilizumab and inflaximab), and recombinant cytokine receptor antagonist proteins (anakinra) [26,602,604,605,606,607,608]. TLR receptor blockers are considered to be promising drugs for the treatment of COVID-19 [51]. However, the drugs, that have already been recommended for use in the treatment of COVID-19 or are undergoing preliminary limited clinical trials, have demonstrated a limited effectiveness, causing an improvement in the condition of patients, their vital signs, and reducing the risk of death [609]. And in some cases, their use not only is inappropriate, but can worsen the course of the disease. 

Therapy with standard doses of dexamethasone slightly reduces the risk of death in patients with COVID-19 who require oxygen support or are on mechanical ventilation, but does not affect the course of the disease and the outcome in those patients who do not receive respiratory support [604]. At the same time, an increase in the doses of dexamethasone not only does not lead to improvement, but on the contrary, is associated with an increased risk of death [610]. Treatment of outpatients with COVID-19 with fluticasone furoate inhalations for 14 days did not have any significant effect on the dynamics of the disease [611]. Inhalation therapy with beclomethasone at the onset of the disease in patients with asymptomatic, mild, or moderate COVID-19 did not affect the progression of the disease to severe COVID-19 [612]. The latest randomized multicenter clinical trial showed that intranasal administration of dexamethasone is more effective than intravenous administration [613]. 

SGLT inhibitors improve metabolism and hemodynamic parameters while possessing anti-inflammatory properties, making them a potential therapeutic agent for the treatment of COVID-19. In particular, they suppress the activity of the inflammasome, which is involved in the pathogenesis of COVID-19 [614,615,616,617]. A retrospective analysis showed that SGLT2 inhibitors reduce the risk of switching to mechanical ventilation in COVID-19 patients with pneumonia [618]. Treatment with the SGLT2 inhibitor dapagliflozin in hospitalized COVID-19 patients with cardiometabolic risk factors reduces the incidence of organ failure or death [619]. However, these observations were not confirmed in later and larger studies. Thus, the randomized, controlled, open-label RECOVERY study did not reveal any beneficial effect of empagliflozin in the treatment of hospitalized COVID-19 patients [620]. Another drug that suppresses inflammasome-mediated inflammation, dimethyl fumarate (DMF), has also been proposed for the treatment of hospitalized COVID-19 patients. Its efficacy was demonstrated in an in vitro SARS-CoV-2 infection model [621], but a randomized, controlled, open-label platform study did not reveal a beneficial effect of DMF on clinical status or any secondary outcome of the disease [622]. 

The emergence of numerous data on the involvement of proinflammatory cytokines in the pathogenesis of COVID-19 allowed us to consider TLR receptors, cytokines, and their receptors as targets for therapy. The selective TLR7/8 inhibitor enpatoran was proposed as one of the drugs with promise for the treatment of COVID-19, but treatment with enpatoran in hospitalized patients with acute COVID-19 pneumonia did not cause significant positive clinical changes and, on average, increased the recovery time compared to patients receiving placebo [623]. Nevertheless, the authors of the study observed potential beneficial effects in preventing disease progression, which confirms the need for the further evaluation of enpatoran. [623]. The addition of infliximab (a monoclonal antibody against TNF-α) to standard therapy contributed to the positive dynamics of the disease and reduced the risk of deterioration in the clinical condition of COVID-19 patients [624]. The use of tocilizumab (a recombinant humanized monoclonal antibody to the human IL-6 receptor) in the treatment of severe COVID-19 patients somewhat reduces the need for mechanical ventilation and the likelihood of death, but is associated with the risk of developing neutropenia and, as a consequence, complications associated with secondary infection [625]. Thus, the use of this drug in the treatment of patients with moderate COVID-19 did not cause any significant positive effect and was associated with the development of secondary infections [626,627]. Treatment with baricitinib reduces mortality in COVID-19, but does not affect the progression of the disease to a severe form requiring mechanical ventilation and ECMO [628]. According to the results of the RECOVERY study, the combined use of corticosteroids, IL-6 receptor blockers, and baricitinib increases the survival of patients with severe COVID-19 [605] and is currently recommended by the World Health Organization for the treatment of such patients. The efficacy of the recombinant interleukin-1 receptor antagonist anakinra in the treatment of COVID-19 remains a matter of debate and further research. On the one hand, there are data demonstrating a positive effect of anakinra on clinical outcomes in patients with COVID-19 [629,630,631,632,633,634,635,636]. On the other hand, the results of some studies show that treatment with anakinra does not affect the survival of patients with COVID-19 [637,638]. 

The use of complement inhibitors in the treatment of COVID-19 has led to controversial results and requires further research. The use of monoclonal antibodies against C5 ravulizumab and zilucoplan did not have any statistically significant effect on the course and outcome of COVID-19 in severe patients [639,640]. On the other hand, the use of the monoclonal antibody against C5a vilobelimab reduced 28-day mortality in critically ill patients receiving invasive mechanical ventilation, with a good safety profile [641]. 

One can see that the use of drugs aimed at suppressing various immune systems and the blood coagulation system demonstrates limited efficacy in the treatment of COVID-19. This may be due to a number of reasons. On the one hand, the pathogenesis of COVID-19 is based on a complex mechanism of interactions between innate and acquired immunity, the blood coagulation system, the fibrinolytic system, and endogenous anticoagulants. At the same time, various elements of these intersystem interactions can both enhance and partially compensate for each other’s functional activity. As a result, suppression of the activity of one link in this interaction network may have a limited effect. On the other hand, the nature of the interactions between the immune and hemostasis systems and the degree of their involvement in the pathogenesis of COVID-19 may change. Thus, the debut of the SARS-CoV-2 infection is accompanied by a sharp increase in the viral load and activation of the innate immune systems [642]. Subsequently, the viral load decreases, and the immune cells, blood coagulation system, endothelial cells, and platelets are involved in the pathogenesis [642]. The blood coagulation system is closely related to the complement system and can enhance its activity, thereby changing the balance between the proinflammatory activities of the complement system and other immune systems, in particular the PRR system. The vascular endothelium and platelets also modulate the proinflammatory response to infection. This, for researchers, raises the question of at what point in the development of COVID-19 the use of a drug aimed at enhancing or inhibiting a specific link in intersystem interactions will be justified and most effective. Thus, the use of interferons, antiviral drugs, and monoclonal antibodies against the SARS-CoV-2 virus is most effective in the first days of the disease [643,644,645]. The inclusion of these drugs in the therapy of hospitalized patients with moderate and severe forms of COVID-19 no longer has a positive effect on the course of the disease and outcome [645,646,647,648,649,650]. It is also important to note that intersystemic interactions in the lungs and in the general bloodstream of COVID-19 patients are different. This may be partly due to the significantly higher viral load in the lungs of COVID-19 patients. This fact dictates the need to find ways to locally modulate the activity of the immune and hemostatic systems to achieve the greatest effectiveness of therapy. Thus, understanding the pathogenetic mechanisms of COVID-19 is necessary to optimize the use of existing therapeutic agents and to find new targets for therapeutic intervention.

## 5. Conclusions

The data presented in the review indicate that COVID-19, like many other infectious diseases associated with the development of thrombotic complications, is a multisystem disease. In particular, the pathogenesis of COVID-19 involves factors of the immune and hemostatic systems, immune cells, vascular endothelium, and platelets, which form a complex network of interactions. Normally, intersystem connections provide a coordinated response to infection, limiting the spread of the pathogen throughout the body and mediating its destruction. However, disruptions in the functioning of one of the elements of this interaction network can lead to disruptions in the functioning of the entire network, involving other elements in pathogenesis and ultimately leading to the development of severe complications and death. Based on the data presented in the literature, we described the mechanisms of intersystem interactions that may be involved in the pathogenesis of COVID-19 and determined the development of complications. When penetrating the body, SARS-CoV-2 activates TLR receptors and the complement system. Both of these systems are involved in activating the immune response to infection and can synergistically interact with each other, enhancing the overall proinflammatory activity. At the same time, SARS-CoV-2 modulates the activity of TLR receptors, suppressing the IFN-mediated response important for fighting the virus and stimulating a cytokine-mediated inflammatory reaction. The inflammatory reaction induced by TLR receptors can be enhanced in several ways at once. On the one hand, the complement system can enhance TLR-mediated production of proinflammatory cytokines. On the other hand, imbalance in differentiation of naive T cells towards proinflammatory Th-17 also contributes to the development of the cytokine-mediated inflammatory response. Both cytokines and complement-activation products can be involved in the activation and damage of the endothelium. As a result, the endothelium acquires procoagulant properties and is involved in the process of the activation of the blood coagulation system and thrombus formation. Since the complement system and the blood coagulation system are closely related, it can be assumed that activation of the complement system can mediate prothrombotic changes in the hemostasis system. In this case, complement factors can directly activate the blood coagulation system, as well as doing so indirectly through the activation of platelets and neutrophils. In turn, the products of the activation of the blood coagulation system can enhance the activity of the complement system, thereby amplifying the inflammatory response. Cytokines can also modulate the activity of the blood coagulation system. In particular, together with the complement system, they can suppress the fibrinolytic system and the endogenous anticoagulant system. This is only part of the range of possible mechanisms that may be involved in the pathogenesis of COVID-19. These assumptions require verification by further studies of the pathogenetic mechanisms of COVID-19. Establishing the exact mechanisms of the pathogenesis of COVID-19 will allow us to develop more effective approaches to treating the disease and reduce the risks of thrombotic complications and other complications.
